# Cytotoxic Properties of 1,3,4-Thiadiazole Derivatives—A Review

**DOI:** 10.3390/molecules25184309

**Published:** 2020-09-20

**Authors:** Sara Janowska, Agata Paneth, Monika Wujec

**Affiliations:** Department of Organic Chemistry, Faculty of Pharmacy, Medical University, 4a Chodzki Str., 20-093 Lublin, Poland; sara.janowska@wp.pl (S.J.); agata.paneth@umlub.pl (A.P.)

**Keywords:** thiadiazole, anticancer activity, cytotoxicity

## Abstract

During recent years, small molecules containing five-member heterocyclic moieties have become the subject of considerable growing interest for designing new antitumor agents. One of them is 1,3,4-thiadiazole. This study is an attempt to collect the 1,3,4-thiadiazole and its derivatives, which can be considered as potential anticancer agents, reported in the literature in the last ten years.

## 1. Introduction

Cancer is one of the major causes of death globally. Based on type and stage of cancer, treatments include radiation therapy, chemotherapy, surgery and targeted therapies. Despite significant advances in medicine, the treatment of cancer remains a serious challenge. Therefore, new, effective drugs and anticancer strategies are still being sought.

One of the methods of searching for new compounds is to modify the structure of known derivatives with documented activity. During recent years, small molecules containing five-membered heterocyclic moieties have become the subject of considerable growing interest for designing antitumor agents. One of them is 1,3,4-thiadiazole. Bioactive properties of thiadiazole are connected generally with the fact, that this heterocyclic ring is a bioisostere of pyrimidine, which in turn is the skeleton of three nucleic bases. Therefore, 1,3,4-thiadiazole derivatives have the ability to disrupt processes related to DNA replication. This permits them to inhibit replication of both bacterial and cancer cells.

Thiadiazole derivatives have four isomeric forms: 1,3,4-thiadiazole; 1,2,3-thiadiazole; 1,2,4-thiadiazole; and 1,2,5-thiadiazole. In published studies, 1,3,4-thiadiazole derivatives tend to show the most significant therapeutic potential [1]. These compounds possessed a wide range of therapeutic activities like antimicrobial [2], antifungal [3], antimycobacterial [4], analgesic, anti-inflammatory [5], antipsychotic [6], antidepressant [7], anticonvulsant [8,9], anti-leishmanial [10]. There are many reports on the antitumor activity of 1,3,4-thiadiazole derivatives.

This study is an attempt to collect the 1,3,4-thiadiazole and its derivatives, which can be considered as potential anticancer agents, reported in the literature between the years 2009 and 2020.

## 2. Derivatives of 2,5-Disubstituted-1,3,4-thiadiazole

**Figure 1:**
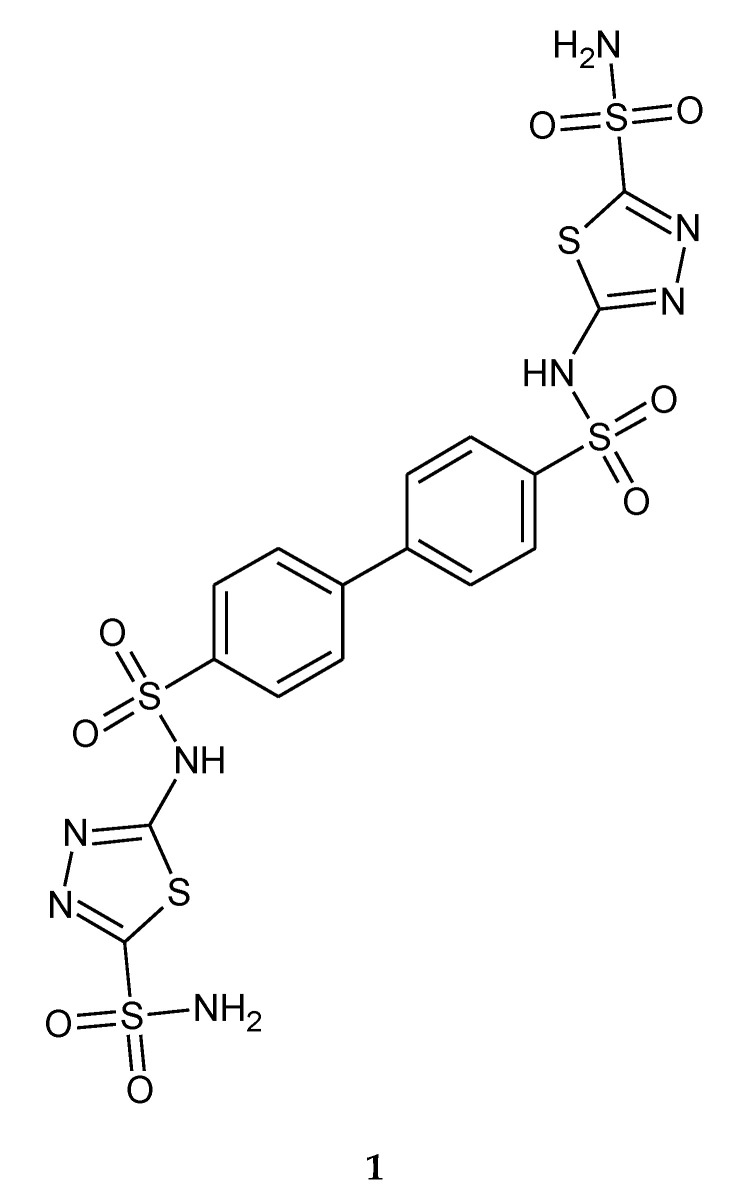
The structure of bis-sulfonamide (**1**).

In 2009, Morsy and co-workers reported anticancer activity in a series of bis-sulfonamides with 1,3,4-thiadiazole heterocyclic rings. The compounds have been tested as inhibitors of CAI, CAII, CAXII and CAIX (carbonic anhydrases). All compounds were rather modest inhibitors of isozymes CA I and XII; but much more efficient as inhibitors of the cytosolic CA II and transmembrane CA IX, with inhibition constants in the range of 21–129 nM against hCA II, and 23–79 nM against hCA IX, respectively. The compounds showed inhibition of growth of several tumor cell lines (ex vivo), with GI_50_ (the concentration causing 50% cell growth inhibition) values in the range of 0.74–10.0 μg/mL against the human colon cancer cell line HCT116, the human lung cancer cell line H460 and the human breast cancer cell line MCF-7. The most active compound (**1**) against the HCT116 cell line and H460 cell lines, with GI_50_ values of 3.29 and 10 μg/mL, respectively, is depicted in Figure 1 [11].

**Figure 2:**
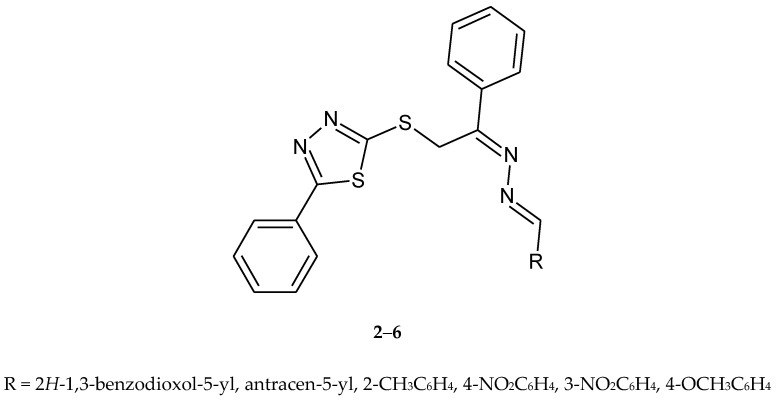
The structures of dihydrazone derivatives (**2**–**6**).

In the same year (2009), Zhang and co-workers reported synthesis and antitumor activity of a series of nine heterocyclic dihydrazone compounds with a 1,3,4-thiadiazole ring (Figure 2). The active compounds **2**–**6** have shown inhibition of growth of the three tumor cell lines: CHO (Chinese hamster ovary), HL60 (human leukemia) and L1210 (mouse leukemia) [12].

**Figure 3:**
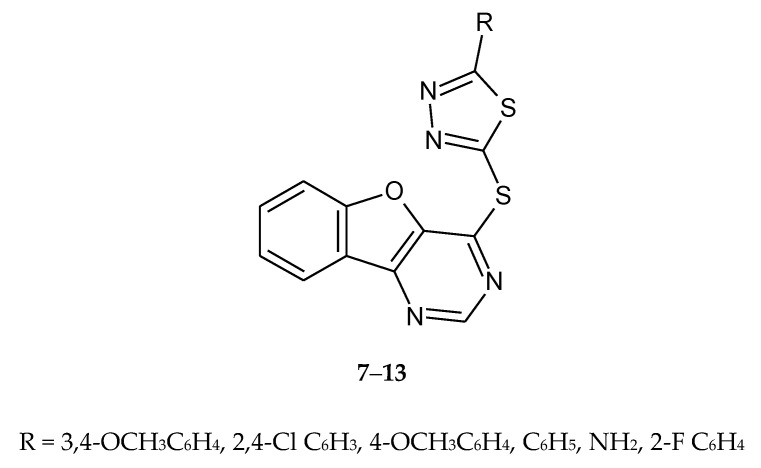
The structures of S-chlorobenzofuro[3,2-d]pyrimidine-1,3,4-thiadiazole (**7**–**13**).

Zhao et al. (2010) designed and synthesized a series of compounds as antitumor agents. The new compounds were prepared by condensation of 1,3,4-thiadiazole-2(3*H*)-thione derivatives and 4-chlorobenzofuro[3,2-d]pyrimidine (Figure 3). The compounds **7**–**13** have shown antiproliferative activities for human prostate tumor cell line PC3. Inhibition of growth in concentration 10 μM/mL was 89.2% [13].

**Figure 4:**
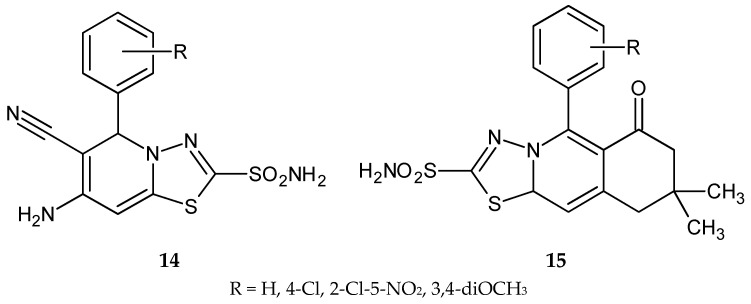
The structures of compounds **14** and **15**.

Synthesis and anticancer activity of a series of new sulfonamide with 1,3,4-thiadiazole heterocyclic rings (Figure 4) were reported by El-Ashmawy and co-workers in 2010. 6-Amino-5-cyano[1,3,4]thiadiazolo[3,2-a]pyrimidine-2-sulfonamide (**14**) and (oxo)[1,3,4]thiadiazolo[2,3-b]quinazoline-2-sulfonamide (**15**) derivatives have shown inhibition of growth activities against the human breast carcinoma (MCF-7) cell line. Anticancer activity of active compounds was higher than bleomycin and ethidium bromide used as reference drugs [14].

**Figure 5:**
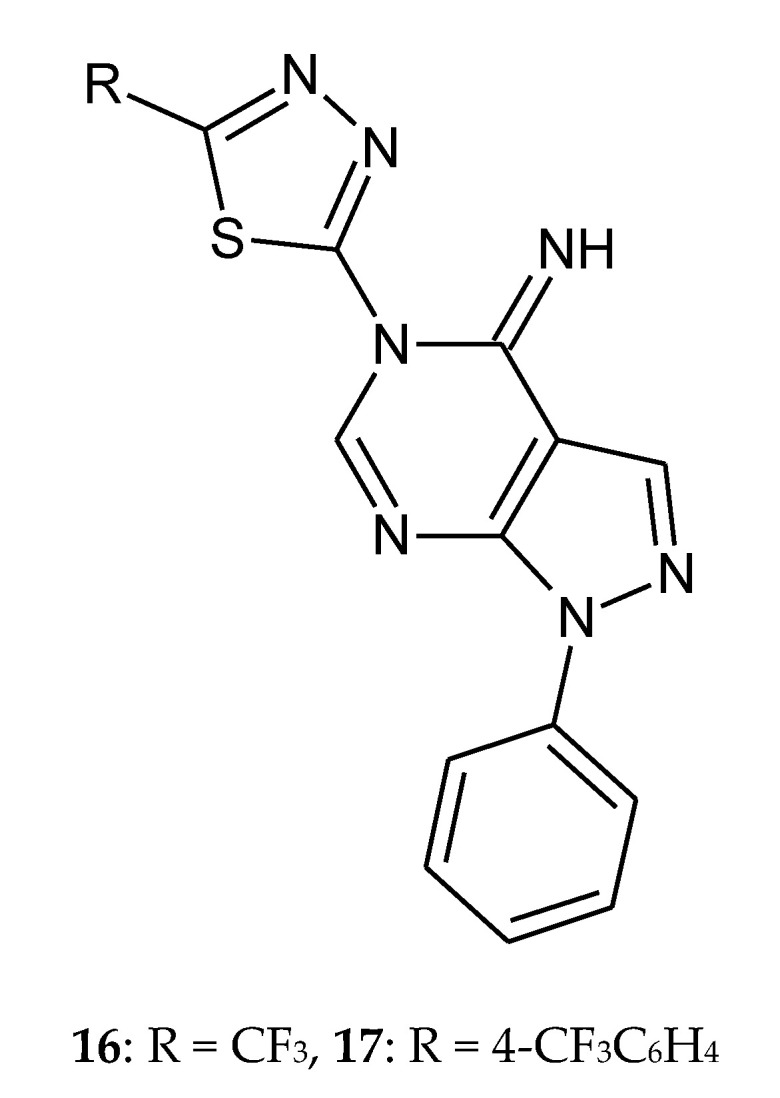
The structures of novel fluorinated pyrazol[3,4-d]pyrimidine with a 1,3,4-thiadiazole heterocyclic ring (**16**–**17**) Table S1.

In 2011, Song et al. reported synthesis and anticancer evaluation of novel fluorinated pyrazolo[3,4-d]pyrimidine with a 1,3,4-thiadiazole heterocyclic ring (Figure 5). The in vitro antitumor activities of the synthesized derivatives against HL-60 (human leukemia cancer cell) were evaluated by the standard MTT assay. Two derivatives with trifluoromethyl (**16**) and 4-trifluoromethylphenyl (**17**) substituent exhibit even higher activity than doxorubicin. Structure-activity relationship analysis indicates that new compounds show better anticancer activity when hydrogen atom in position 1 of the pyrazole ring is substituted with a phenyl group. Additionally, incorporation of the CF_3_ group in the molecules increases biological activity significantly [15].

**Figure 6:**
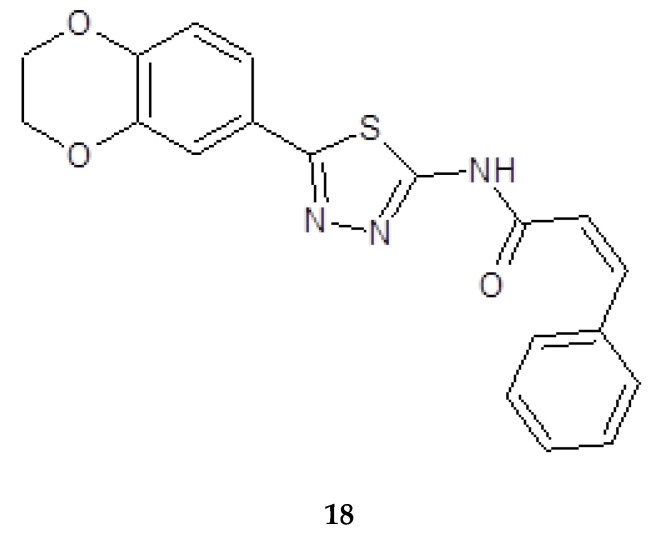
The structure of *N*-(5-(2,3-dihydrobenzo[b][1,4]dioxin-6-yl)-1,3,4-yl)cinnamamide (**18**).

Synthesis, molecular docking studies and anticancer evaluation of the novel 1,3,4-thiadiazole derivatives containing 1,4-benzenodioxan was described by Sun and co-workers in 2011. All the synthesized derivatives were evaluated for their antiproliferative ability against HEPG2, HELA, SW1116 and BGC823. The cinnamamide derivatives (**18**) (Figure 6) showed the most potent biological activity against HEPG2 cancer cell line with EC_50_ value of 10.28l g/mL.

The authors selected compounds to test their FAK (focal adhesion kinase) inhibitory activity against HEPG2 cell line. Most of the tested compounds displayed potent FAK inhibitory. The results of FAK inhibitory activity of the tested compounds were corresponding to the structure relationships of their antitumor activities. In docking simulation for FAK, the compound **18** also showed good effects (EC_50_ = 10.79 μM, half maximal effective concentration) [16].

**Figure 7:**
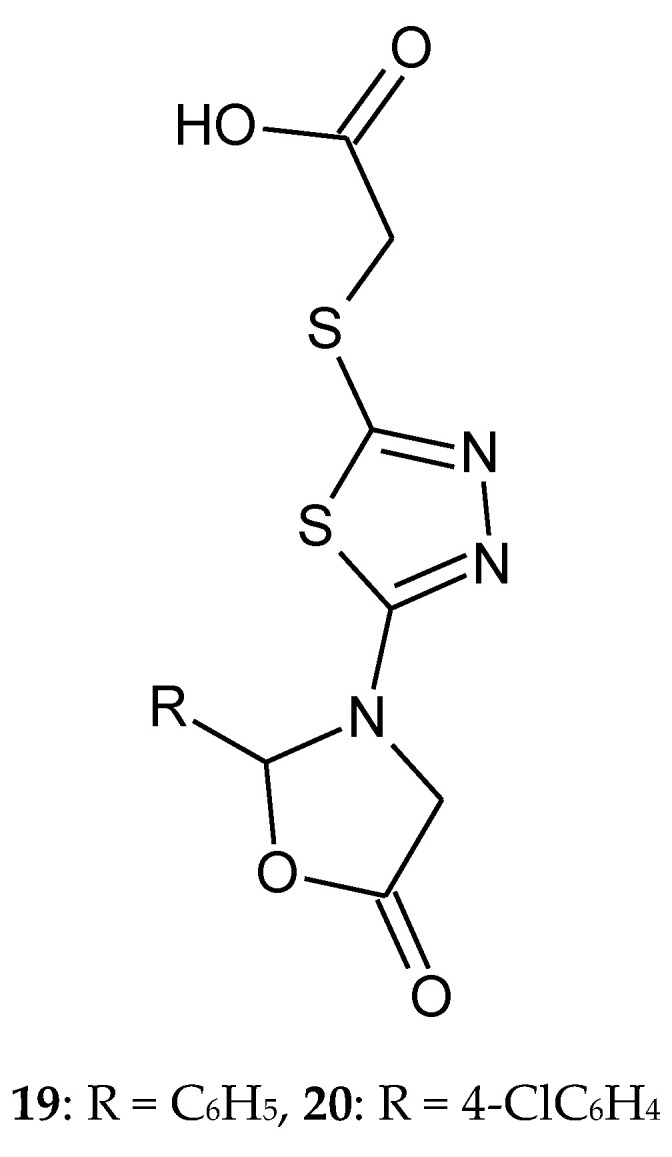
The structures of compounds **19** and **20**.

In 2011, El-Naggar and co-workers reported synthesis and anticancer evaluation of series 1,3,4-thiadiazole and 1,2,4-triazine. All compounds indicated Ehrlichs Ascites Carcinoma cells (EAC) intra-peritoneal (i.p.) (2 × 106 cells mouse-1) after in vivo testing. 1,3,4-Thiadiazole derivatives inhibited tumor growth after 14 days of the treatment. 1,2,4-triazine compounds have not shown any anticancer activity against EAC-model. 2-(5-(5-Oxo-2-phenyloxazolidin-3-yl)-1,3,4-thiadiazol-2-ylthio)acetic acid (**19**) and 2-(5-(5-oxo-2-(4-chlorophenyl)oxazolidin-3-yl)-1,3,4-thiadiazol-2-ylthio)acetic acid (**20**) have shown the best antitumor activity (Figure 7) [17].

**Figure 8:**
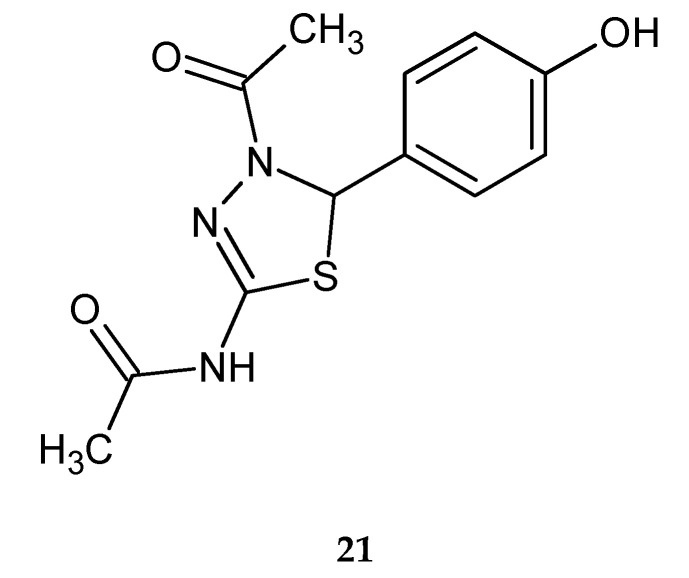
The structure of N-(4-acetyl-5-(4-hydroxyphenyl)-4,5-dihydro-1,3,4-thiadiazol-2-yl)-acetamide (**21**).

Alam et al. (2011) reported synthesis and anticancer evaluation of a series of 5-phenyl-4,5-dihydro-1,3,4-thiadiazoles. Most of the tested compounds have shown significant suppressive activity against the growth of all of the human cancer cell lines: lung cancer (A549), skin cancer (SK-MEL-2), ovarian cancer (SK-OV-3) and colon cancer (HCT15). *N*-(4-acetyl-5-(4-hydroxyphenyl)-4,5-dihydro-1,3,4-thiadiazol-2-yl)-acetamide (**21**) (Figure 8) was most active in the inhibition of growth of the SK-MEL-2 cell line, with an IC_50_ value (the half maximal inhibitory concentration) of 4.27 µg/mL. A structure–activity relationship study revealed that the nature of substituent on the C-5 phenyl ring of 1,3,4-thiadiazoles is important for their cytotoxic activity [18].

**Figure 9:**
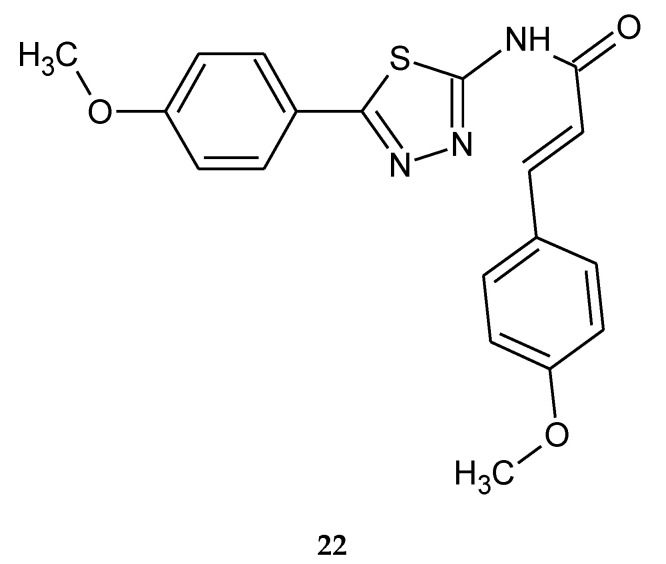
The structure of (*E*)-3-(4-methoxyphenyl)-*N*-(5-(4-methoxyphenyl)-1,3,4-thiadiazol-2-yl)acrylamide (**22**).

Anticancer activity of a series of cinnamic acid with a 1,3,4-thiadiazole heterocyclic ring was reported by Yang (2012). The compound with two methoxy groups in phenyl rings **22** (Figure 9) has shown the best activity against breast cancer (MCF-7) and lung carcinoma (A549) cell lines, with IC_50_ values of 0.28 and 0.52 μg/mL. Additionally, the authors investigated the inhibitor’s interaction with tubulin by docking study. After analysis of the binding model of compound **22** with tubulin (IC_50_ = 1.16 μg/mL), it was found that two hydrogen bonds and two π-cation interactions with the protein residues in the colchicine binding site might play an important role in its antitubulin polymerization and antiproliferative activities [19].

**Figure 10:**
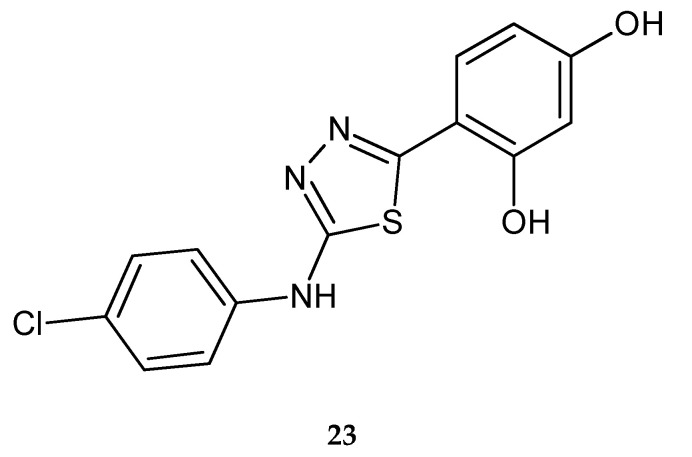
The structure of 2-(4-chlorophenylamino)-5-(2,4-dihydroxyphenyl)-1,3,4-thiadiazole (**23**).

The synthesis and anticancer activity of 2-(4-chlorophenylamino)-5-(2,4-dihydroxyphenyl)-1,3,4-thiadiazole was reported by Juszczak et al. (2012). The new compound **23** (Figure 10) has shown good antiproliferative activity against peripheral cancers including breast carcinoma (T47D), colon carcinoma (HT-29), thyroid carcinoma(FTC-238), teratoma (P19), and T-cell leukemia (Jurkat E6.1), as well as cancers of the nervous system including rhabdomyosarcoma/medulloblastoma (TE671), brain astrocytoma (MOGGCCM) and glioma (C6). The in vitro tests were carried out by using the MTT assay. Compound **23** was not toxic for normal cells: skin fibroblasts, hepatocytes, astrocytes and neurons [20].

**Figure 11:**
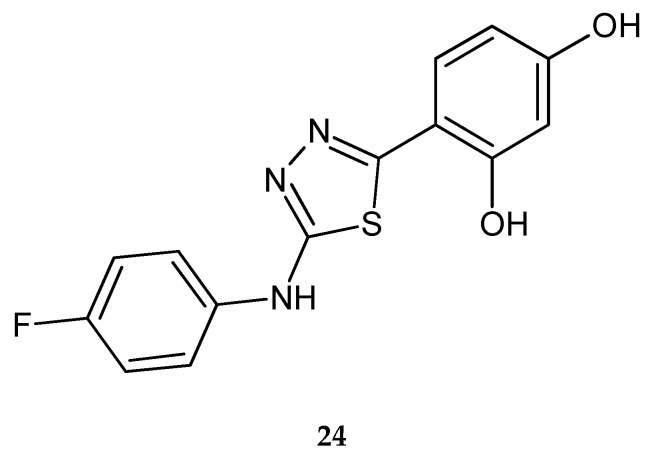
The structure of 2-(4-fluorophenyloamino)-5-(2,4-dihydroxyphenyl)-1,3,4-thiadiazole (**24**).

The same authors in 2012 reported anticancer activity of 2-(4-fluorophenyloamino)-5-(2,4-dihydroxyphenyl)-1,3,4-thiadiazole (**24**) (Figure 11). Compound has shown good antiproliferative activity against A549 lung carcinoma cells. Replacing of the chlorine atom with a fluorine atom causes an increase in anticancer activity. Western blotting analysis has shown that activity of compound **24** is connected with inhibition of kinase 1/2 (ERK1/2). The new compound induces cell cycle progression through G1 into S phase in cells [21].

**Figure 12:**
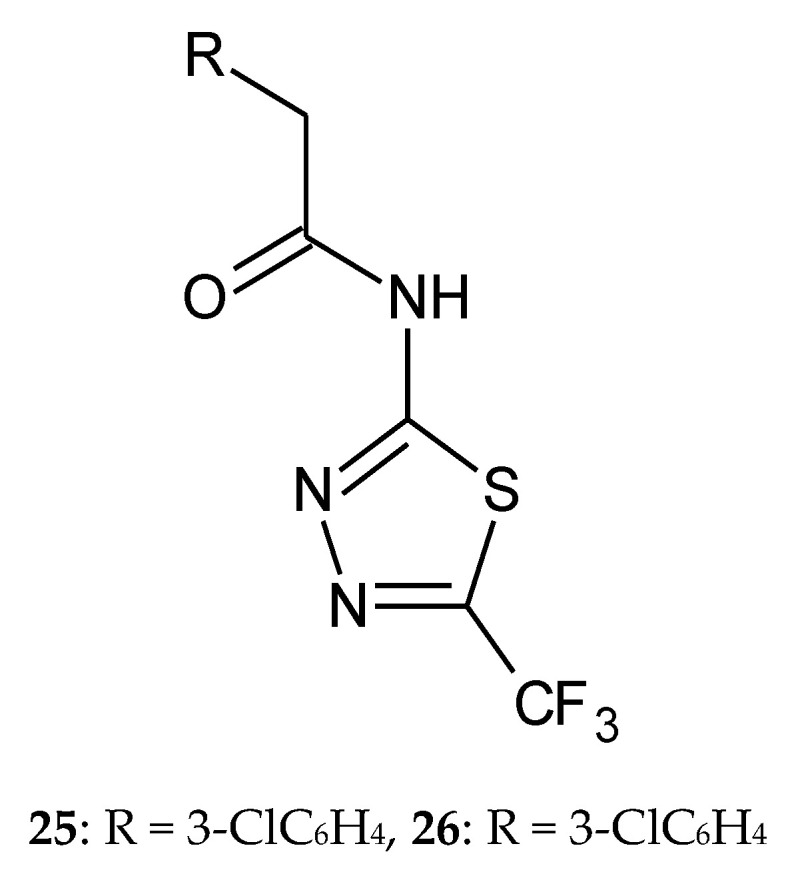
The structures of 2-(3-chlorophenyl)-*N-*(5-(trifluoromethyl)-1,3,4-thiadiazol-2-yl)acetamide (**25**) and 2-(4-chlorophenyl)-*N-*(5-(trifluoromethyl)-1,3,4-thiadiazol-2-yl)acetamide (**26**).

In 2013, Hosseinzadeh Leila et al. described synthesis and anticancer activity of a new 1,3,4-thiadiazole with trifluoromethyl substituent. The derivatives were evaluated for their anticancer activity against three cancer cell lines: PC3 (prostate cancer), MCF7 (breast cancer), and SKNMC (neuroblastoma) in the MTT assay. Doxorubicin was used as reference drug. Two of the synthesized compounds—with 3- (**25**) and 4-chloro (**26**) (Figure 12) substituents—have shown better cytotoxic activity than others for MCF7 cell line. The authors investigated apoptosis induction through exploration of the activation of caspases 3, 8, and 9. According to the obtained results, compounds **25** and **26** demonstrated the best caspase activation [22].

**Figure 13:**
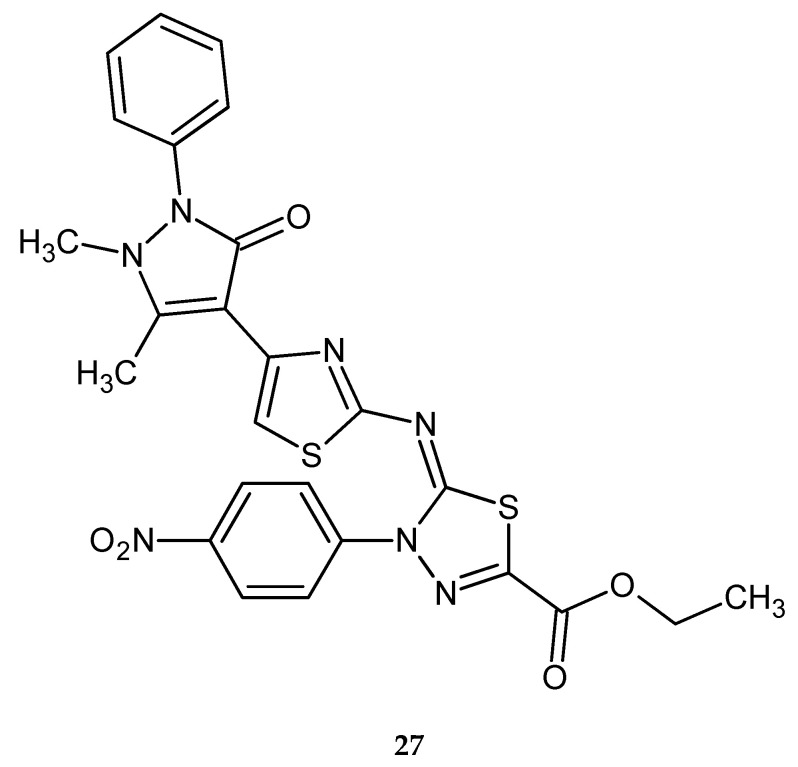
The structure of ethyl 2-(4-(2,3-dimethyl-1-phenyl-5-oxo-pyrazol-4-yl)thiazol-2-ylimino)-3-(4-nitrophenyl)-1,3,4-thiadiazole-5-carboxylate (**27**).

Synthesis and anticancer activity of a series of new pyrazole-based 1,3-thiazoles and 1,3,4-thiadiazoles were reported by Dawood and co-workers in 2013. The compounds were evaluated for their anticancer activity against human hepatocellular carcinoma HepG2, human breast cancer MCF-7 and human lung cancer A549. Most of them have shown moderate-to-high activity, with the best IC_50_ value of 8.107 μM (HepG2), 10-times higher than for doxorubicin (0.877 μM) for compound with 4-nitrophenyl substituent **27** (Figure 13) [23].

**Figure 14:**
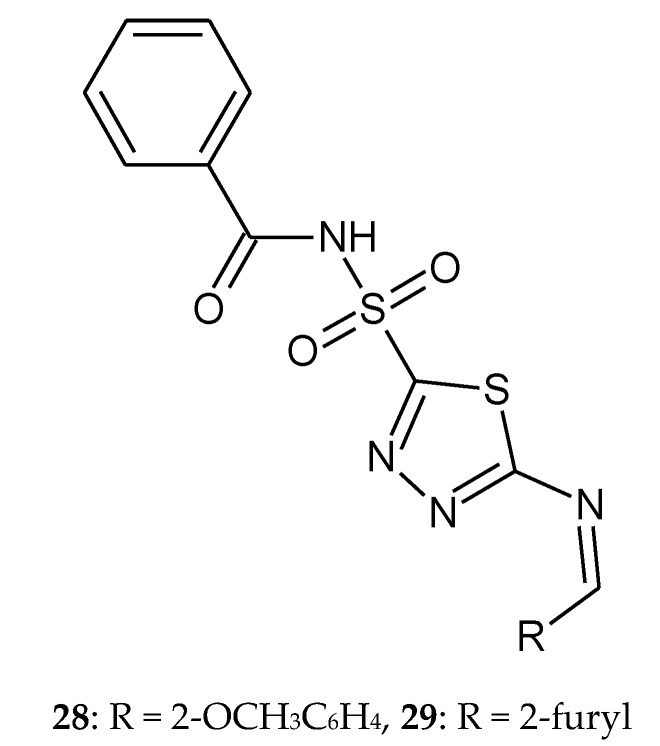
The structures of the most active compounds **28** and **29**.

In 2014, Chhajed and co-workers reported synthesis of 5-arylidineamino-1,3,4-thiadiazol-2-[(*N*-benzoyl)] sulfonamide derivatives. New compounds were obtained from carbonic anhydrase inhibitor drug acetazolamide. All the synthesized compounds were investigated for anticancer activity towards cell lines HEK 293 (epidermal kidney cell line), BT474(breast cancer cell line) and NCI-H226 (lung cancer). The results were compared with standard drug indisulam (ISL). The results demonstrated a strong dose-dependent growth inhibition intreated cell lines. The most active compounds are: *N*-({5-[(2-methoxybenzylidene)amino]-1,3,4-thiadiazol-2-yl}sulfonyl)benzamide **28** (CTC_50_ = 0.794 µM against breast cancer cell line, CTC_50_—concentration required to reduce viability by 50%) and *N*-({5-[(furan-2-ylmethylidene)amino]-1,3,4-thiadiazol-2-yl}sulfonyl)benzamide **29** (CTC_50_ = 0.913 µM against lung cancer cell line) (Figure 14). None of the tested compounds were found to be as potent as the reference drug [24].

**Figure 15:**
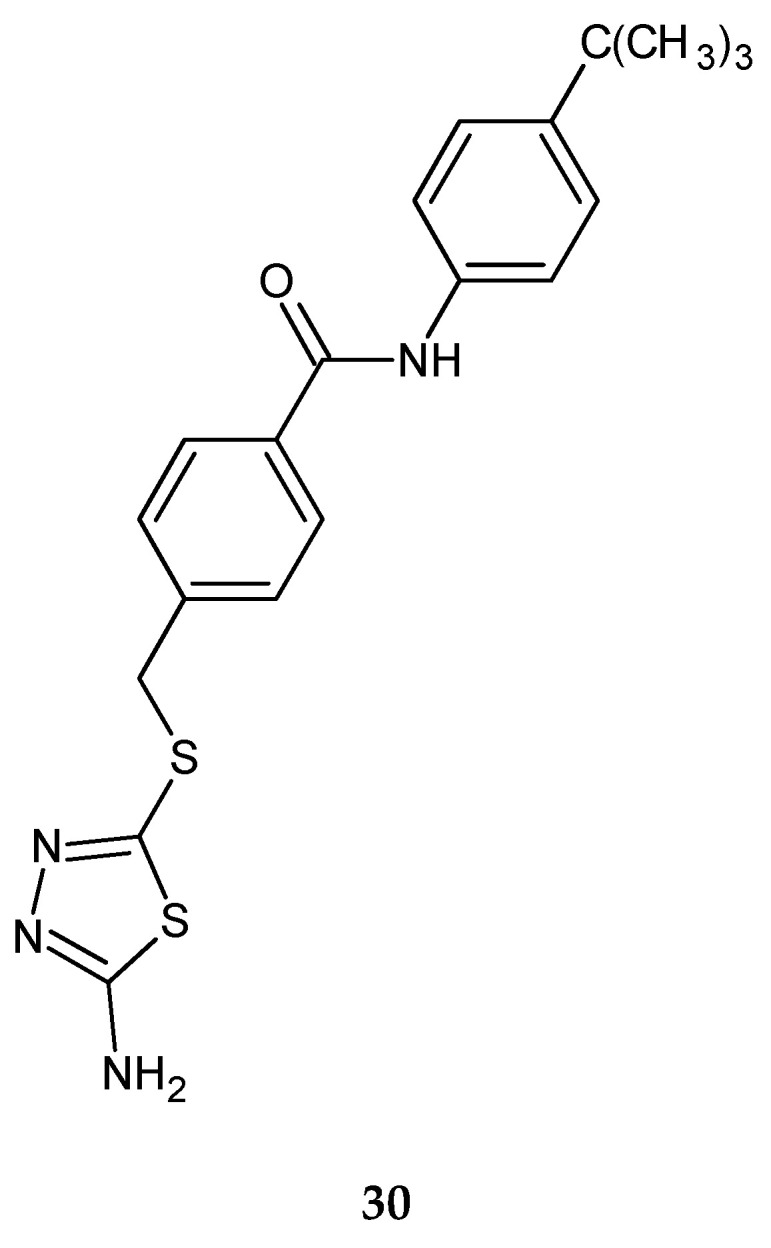
The structure of 4-[(5-amino-1,3,4-thiadiazol-2-ylthio)methyl]-*N*-(4-*tert*-butylphenyl) benzamide (**30**).

Shi et al. (2013) reported synthesis and anticancer activity of a new 1,2,4-triazole and 1,3,4-thiadiazole. The cytotoxicity of the synthesized compounds were tested on five human tumor cell lines, including human pancreatic cancer cell line (BxPC-3), human lung adenocarcinoma cell line (H1975), human ovarian carcinoma cell line (SKOV-3), human melanoma cell line (A875) and human colorectal adenocarcinoma cell line (HCT116) by the MTT assay with adriamycin as a positive control. Some compounds have shown good activity in the test, with IC_50_ values from 0.04 to 23.6 µM. 1,2,4-triazole derivatives exhibited much better potency than 1,3,4-tiadiazole analogs. The most active thiadiazole derivative was with *tert*-butyl substituent (IC_50_ value 1.7 µM against human pancreatic cancer cell line) **30** (Figure 15) [25].

**Figure 16:**
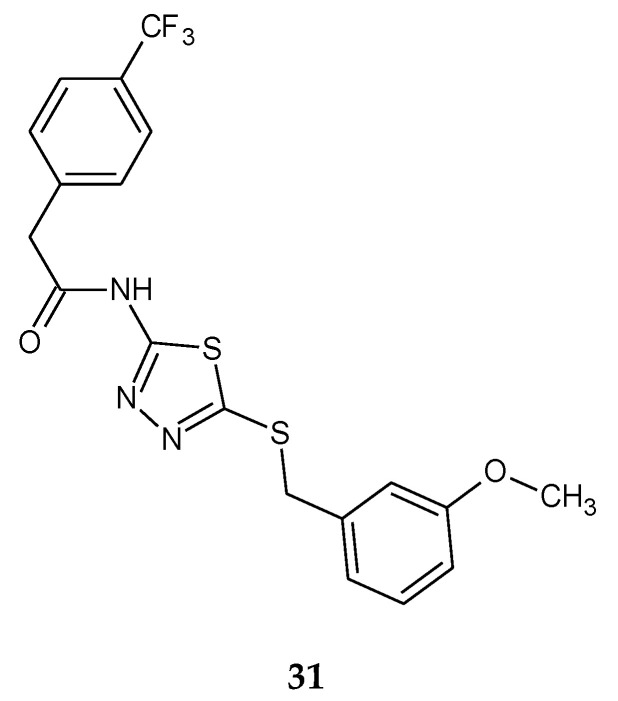
The structure of *N*-(5-(3-methoxybenzylthio)-1,3,4-thiadiazol-2-yl)-2-(4-(trifluoromethyl)phenyl)acetamide (**31**) (Table S1).

The synthesis and anticancer activity of a series of *N*-(5-(benzylthio)-1,3,4-thiadiazol-2-yl)-2-(4-(trifluoromethyl)phenyl)acetamide were reported by Aliabadi in 2013. The compounds were evaluated for their anticancer activity against three human cancers: prostate (PC3), breast (MDA) and glioblastoma (U87). The best activity was observed against MDA breast cancer cell line. Compound **31** (Figure 16) showed higher activity with IC_50_ = 9 μM compared to Imatinib (IC_50_ = 20 μM) in MDA breast cancer cell line [26].

**Figure 17:**
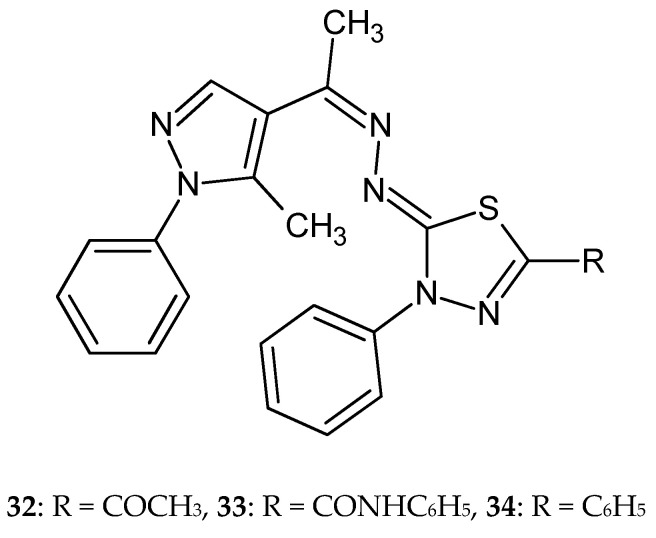
The structures of 2-[[1-(5-methyl-1-phenyl-5-substituted-1*H*-pyrazol-4-yl)-ethylidene]hydrazono]-3-phenyl-2,3-dihydro-1,3,4-thiadiazoles (**32**–**34**).

In 2015, Gomha et al. reported anticancer activity in a series of thiadiazoles and thiazoles. The compounds were evaluated for their anticancer activity against the breast carcinoma cell line MCF-7. The compounds have shown good inhibitory effect in test with IC_50_ values of 21.3 ± 0.72, 22.65 ± 0.72, and 23.56 ± 0.81 μg/mL for most active derivatives **32**–**34** (Figure 17). Confocal laser scanning imaging of the treated cells stained by rhodamin 123 and acridine orange dyes showed that some compounds inhibit the mitochondrial lactate dehydrogenase enzymes [27].

**Figure 18:**
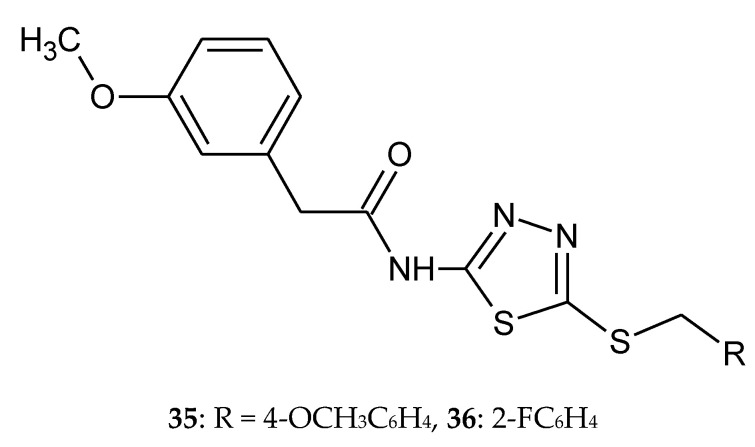
The structures of *N*-(5-(4-methoxybenzylthio)-1,3,4-thiadiazol-2-yl)-2-(3-methoxyphenyl)acetamide (**35**) and *N*-(5-(2-fluorobenzylthio)-1,3,4-thiadiazol-2-yl)-2-(3-methoxyphenyl)acetamide (**36**).

The synthesis, docking study and cytotoxicity evaluation of *N*-(5-(benzylthio)-1,3,4-thiadiazol-2-yl)-2-(3-methoxyphenyl)acetamide derivatives, such as tyrosine kinase inhibitors, were reported by Mohammadi-Farani et al. (2014). Anticancer activity was estimated against human prostate cancer (PC3), human neuroblastoma (SKNMC) and human colon cancer (HT29) cell lines, by MTT assay. Compound with methoxy group in position *para* **35** (Figure 18) demonstrated the highest inhibitory potency against PC3 (IC_50_ = 22.19 ± 2.1 µM) and SKNMC (IC_50_ = 5.41 ± 0.35 µM) cell lines. This compound showed better cytotoxic activity than Imatinib used as reference drug. Compound with fluorine in position *ortho* **36** (Figure 18) exhibited the most cytotoxic activity against HT29 cell line with IC_50_ value 12.57 ± 0.6 µM. Molecular docking studies demonstrated potential hydrogen bindings for ligand–receptor interaction [28].

**Figure 19:**
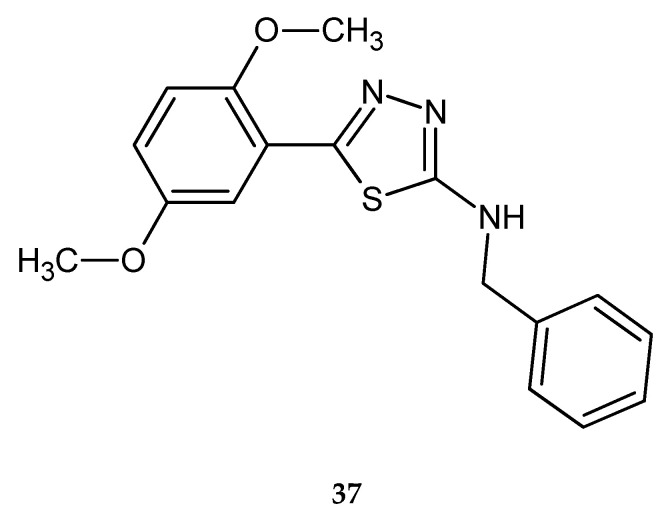
The structure of 2-(*N*-benzyl-amine)-[5-(2,5-dimethoxy-phenyl)-1,3,4-thiadiazole (**37**).

In 2015, Polkam and co-workers reported synthesis, in vitro anticancer and antimycobacterial activity of 5-(2,5-dimethoxy-phenyl)-2-substituted-1,3,4-thiadiazole. Anticancer activity was tested against HT-29 colon cancer and MDA-MB-23 breast cancer cell lines, by MTT assay. Some compounds have shown good inhibitory effect in the test, with the best cytotoxicity inhibition of 68.28% (HT-29) and 62.95% (MDA-MB-23) for derivatives with benzyl substituent **37** (Figure 19). The derivatives were also tested on human normal cell line and they exhibited selectivity for cancer cells in that test [29].

**Figure 20:**
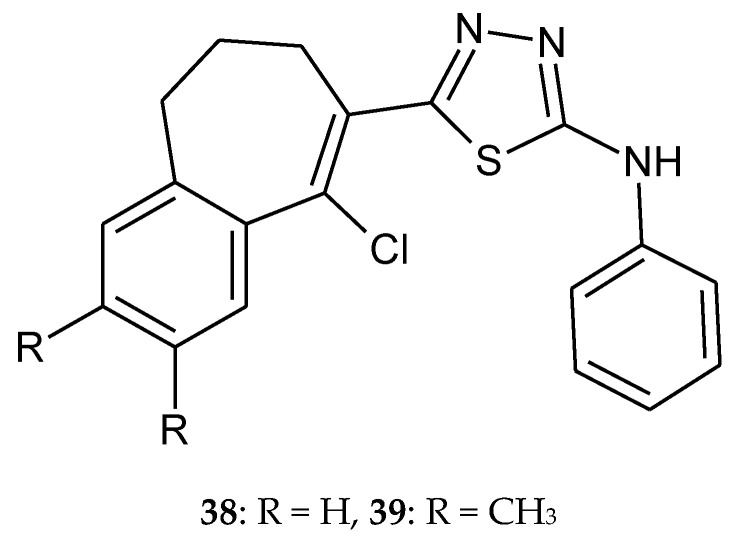
The structures of 5-(9-chloro-6,7-dihydro-5*H*-benzo[7]annulen-8-yl)-2-(*N*-phenylamino)-1,3,4-thiadiazole (**38**) and 5-(9-chloro-2,3-dimethyl-6,7-dihydro-5*H*-benzo[7]annulen-8-yl)-2-(*N*-phenylamino)-1,3,4-thiadiazole (**39**).

Yadagiri et al. (2015) described synthesis and anticancer activity the series of 1,3,4-oxadiazole, 1,3,4-thiadiazole and 1,2,4-triazole with benzoannulen scaffold. The compounds were evaluated for their anticancer activity against HeLa, breast cancer MDA MB 231, human pancreatic cancer PANC1 and lung cancer A549 cell lines, by SRB assay. Most of the novel compounds showed significant antiproliferative activity against four different human cancer cell lines with GI_50_ values in the range of 0.079–8.284 µM. Oxadiazoles were more active than thiadiazoles **38**–**39** (Figure 20) [30].

**Figure 21:**
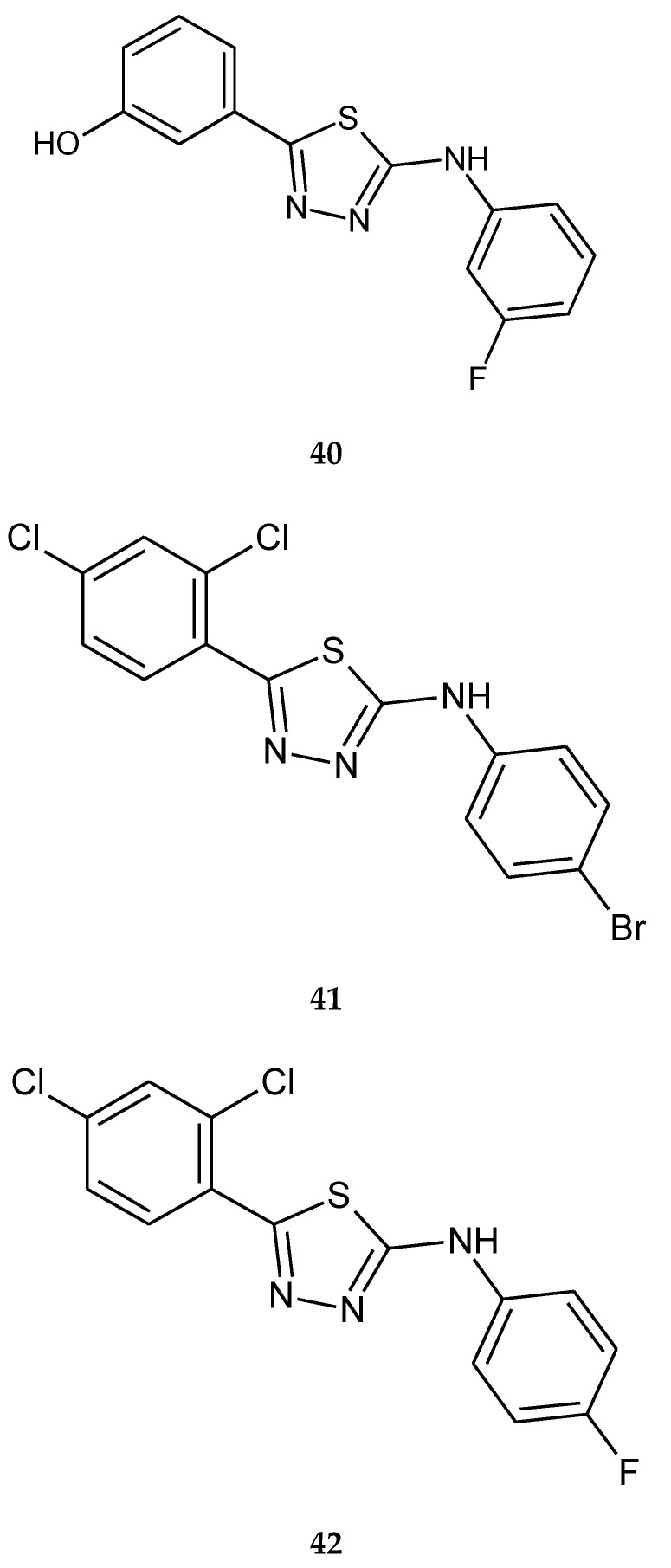
The structures of compounds (**40**–**42**).

Plech et al. (2015) reported anticancer activity of a series of 2,5-disubstituted-1,3,4-thiadiazole. The compounds were evaluated for their anticancer activity against human breast cancer MCF-7 and MDA-MB-231 cell lines, by MTT assay. 2-(3-Fluorophenylamino)-5-(3-hydroxyphenyl)-1,3,4-thiadiazole (**40**), 2-(4-bromophenylamino)-5-(2,4-dichlorophenyl)-1,3,4-thiadiazole (**41**), 2-(4-fluorophenylamino)-5-(2,4-dichlorophenyl)-1,3,4-thiadiazole (**42**) (Figure 21) have shown ability to inhibit proliferation cancer cells. The compounds have shown moderate antiproliferative activity with IC_50_ values from 120 to 160 μM (MCF-7) and from 70 to 170 μM (MDA-MB-231) [31].

**Figure 22:**
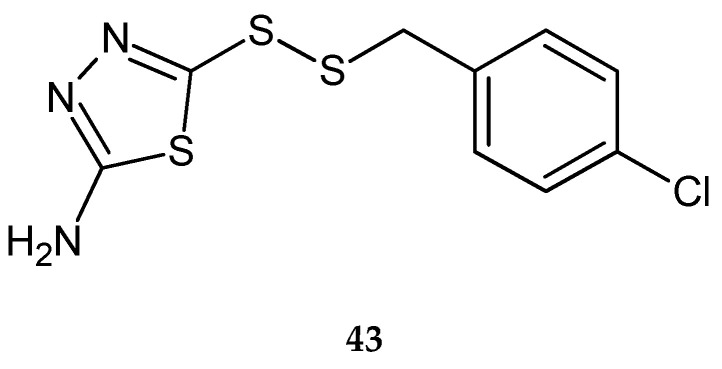
The structure of 4-chlorobenzyl-(2-amino-1,3,4-thiadiazol-5-yl)disulfide (**43**) (Table S1).

Li et al. (2015) reported synthesis and antitumor activity of disulfide derivatives containing a 1,3,4-thiadiazole moiety. They were evaluated anticancer activity against human cell lines: breast cancer MCF-7, hepatocarcinoma SMMC-7721 and lung cancer A549, by CCK-8 assay. The compounds have shown better antiproliferative activity then 5-fluorouracil used as reference drug. 4-Chlorobenzyl-(2-amino-1,3,4-thiadiazol-5-yl)disulfide (**43**) (Figure 22) compounds have shown significant inhibition of proliferation activities against breast and lung cancer with IC_50_ = 1.78 μmol/L and IC_50_ = 4.04 μmol/L, respectively [32].

**Figure 23:**
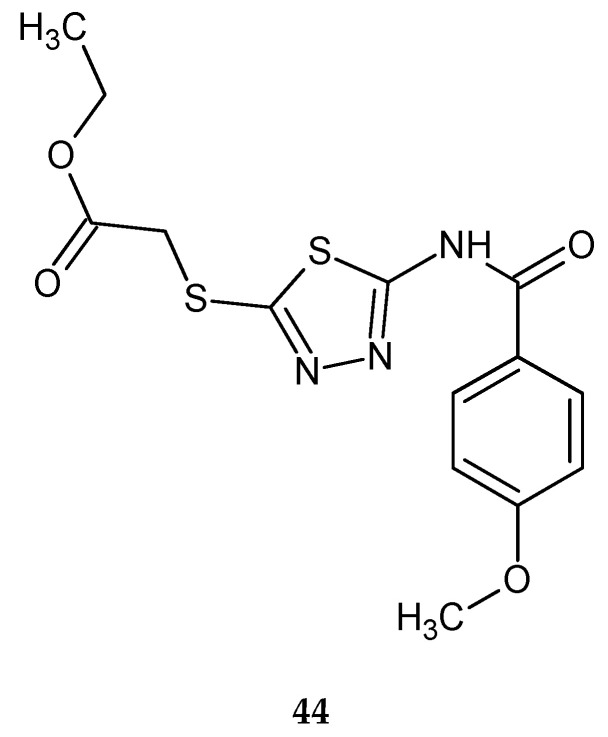
The structure of ethyl 2-((5-(4-methoxybenzamido)-1,3,4-thiadiazol-2-yl)thio)acetate (**44**).

In 2016, Almasirad and co-workers described synthesis and biological evaluation of new series of 2-amido-1,3,4-thiadiazole derivatives as cytotoxic agents. The compounds were evaluated for their anticancer activity against human leukemia HL-60, human ovarian cancer SK-OV-3 and human acute T lymphoblastic leukemia MOLT-4 cell lines, by MTT assay. The compound with 4-methoxyphenyl substituent **44** (Figure 23) has shown the best anticancer activity against SK-OV-3 cell line with IC_50_ = 19.5 μM [33].

**Figure 24:**
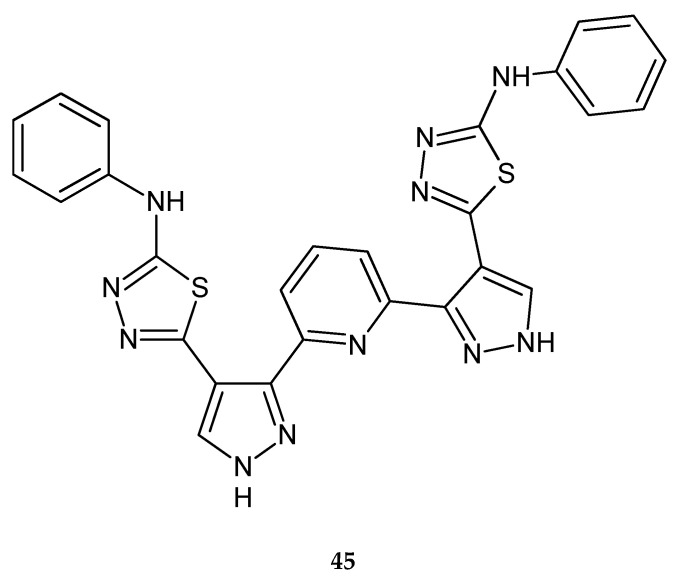
The structure of 2,6-bis[4-(5-phenylamino-1,3,4-thiadiazol-2-yl)-1*H*-pyrazol-3-yl]pyridine (**45**).

Synthesis and antitumor screening of some new 2,6-bis pyridines functionalized with pyrazole-based heterocycles were described by Ali et al. in 2015. Some of the compounds had a 1,3,4-thiadiazol ring. The newly synthesized compounds were screened for their in vitro cytotoxic activity against three human tumor cell lines such as human lung adenocarcinoma (A549), hepatocellular carcinoma (HEPG2) and breast adenocarcinoma (MCF7). Thiadiazole derivative **45** (Figure 24) was not active; it had a cytotoxic activity percentage less than 10% [34].

**Figure 25:**
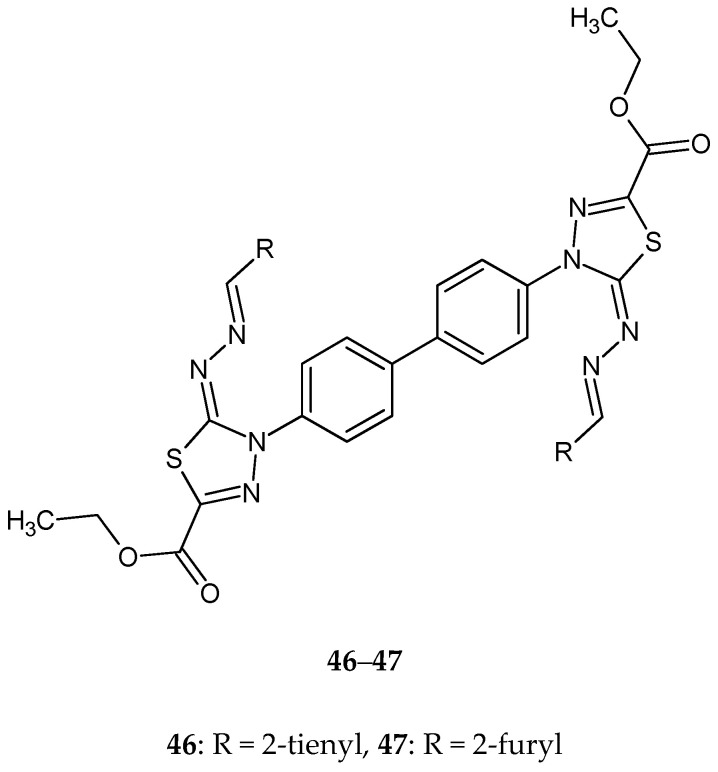
The structures of diethyl 4,4’-(biphenyl-4,4’-diyl)bis(5-((thiophen-2-ylmethylene)hydrazono)-4,5-dihydro-1,3,4-thiadiazole2-carboxylate (**46**) and diethyl 4,4’-(biphenyl-4,4’-diyl)bis(5-((furan-2-ylmethylene)hydrazono)-4,5-dihydro-1,3,4-thiadiazole-2-carboxylate) (**47**).

**Figure 26:**
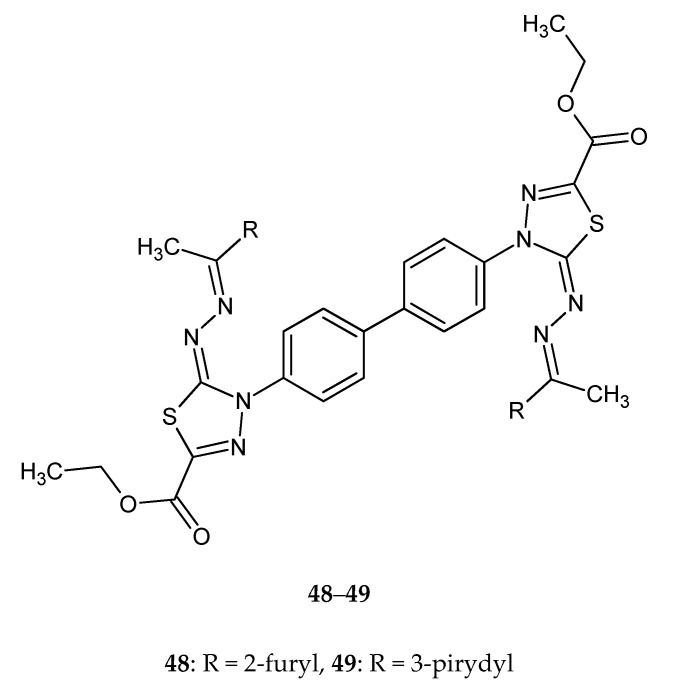
The structures of diethyl4,4’-(biphenyl-4,4’-diyl)bis(5-((1-(furan-2-yl)ethylidene)hydrazono)-4,5-dihydro-1,3,4-thiadiazole-2-carboxylate) (**48**) and diethyl 4,4’-(biphenyl-4,4’-diyl)bis(5-((1-(pyridin-3-yl)ethylidene)hydrazono)-4,5-dihydro-1,3,4-thiadiazole-2-carboxylate) (**49**).

Gomha and co-workers (2016) reported synthesis and biological evaluation of some novel bis(1,3,4-thiadiazole)derivatives as potential cytotoxic agents. The compounds were evaluated for their anticancer activity against human breast cancer MCF-7 cell lines, by MTT assay. Derivatives **46**–**47** (Figure 25) with 2-thienyl or 2-furyl substituent and compounds **48**–**49** with 2-furyl or 3-pyridyl (Figure 26) substituent exhibited promising anticancer activities, with activity better than imatinib [35].

**Figure 27:**
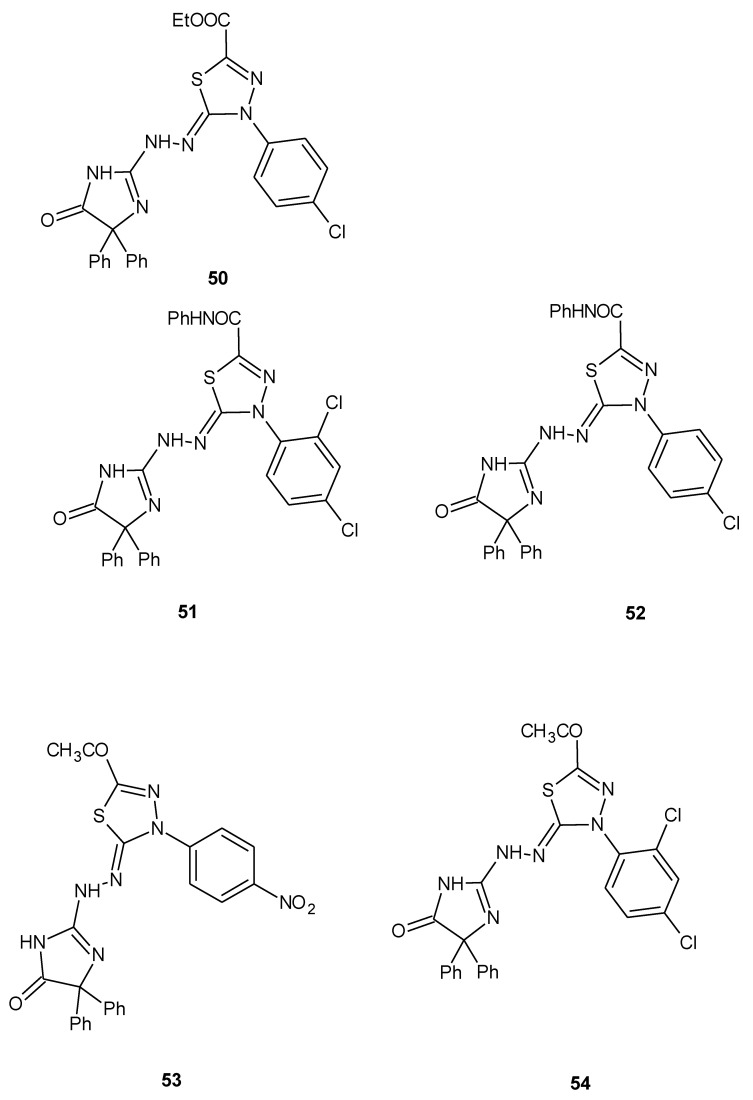
The structures of the most active imidazole–thiadiazole derivatives (**50**–**54**).

The same authors reported synthesis and SAR study of the novel imidazole–thiadiazole derivatives and their activity. The scientists used liver carcinoma cell line HEPG2-1. The results indicated that some of the tested compounds showed moderate-to-high cytotoxic activity with respective to doxorubicin used as a reference drug. The compounds **50**–**54** (Figure 27) exhibited promising anticancer activities, with IC_50_ = 0.86, 1.02, 1.17, 1.08, 1.44 μM, respectively [36].

**Figure 28:**
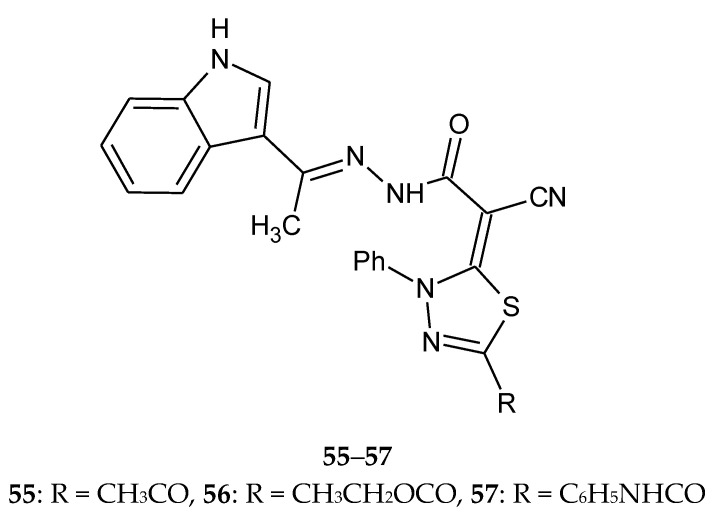
The structure of *N*’-(1-(1*H*-indol-3-yl)ethylidene)-2-(5-R-3-phenyl-1,3,4-thiadiazol-2(3H)-ylidene)-2-cyanoaceto-hydrazide (**55**–**57**).

In the same year (2016), Abdelhamid et al. described synthesis of new 3-heteroarylindoles as potential anticancer agents. Some of 1,3,4-thiadiazole derivatives were obtained in the reaction of hydrazonoyl halides with *N*’-(1-(1H-indol-3-yl)ethylidene)-2-cyanoacetohydrazide. The new compounds were evaluated for their antitumor activity against the MCF-7 human breast carcinoma cell line. The results indicated that many of the tested compounds showed moderate-to-high anticancer activity. The IC_50_ values for thiadiazole derivatives **55**–**57** (Figure 28) were in the range 1.01–2.04 µM (IC_50_ for doxorubicin was 0.75 µM) [37].

**Figure 29:**
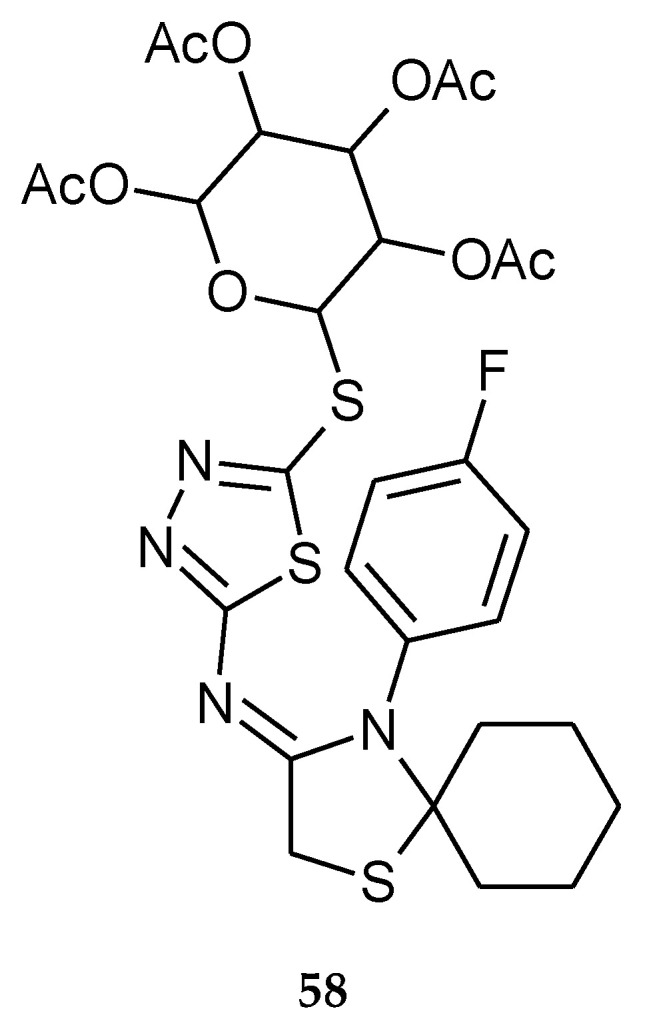
The structure 4-(4-fluorophenyl)-*N*-(5-(2,3,4,6-tetra-*O*-acetyl-D-glucopyranosyl)-1,3,4-thiadiazol-2-yl)-1-thia-4-azaspiro[4.5]decan-3-imine (**58**) (Table S1).

Flefel and co-workers reported anticancer activity of a series of 1,3,4-thiadiazole thioglycosides (2017). For their study they used human liver hepatocellular carcinoma (HepG-2), human prostate adenocarcinoma (PC-3) and human colorectal carcinoma (HCT-116) cell lines. The compounds have shown moderate-to-good anticancer activity during in vitro tests. The results revealed that the attachment of glycosyl moieties to the 1,3,4-thiadiazole ring is very important to activity in this class of compounds. Compound **58** (Figure 29) showed good anticancer activity against HCT-116 carcinoma cells with IC_50_ = 92.2 ± 1.8 nM while IC_50_ for doxorubicin it was 126.7 ± 8.9 nM [38].

**Figure 30:**
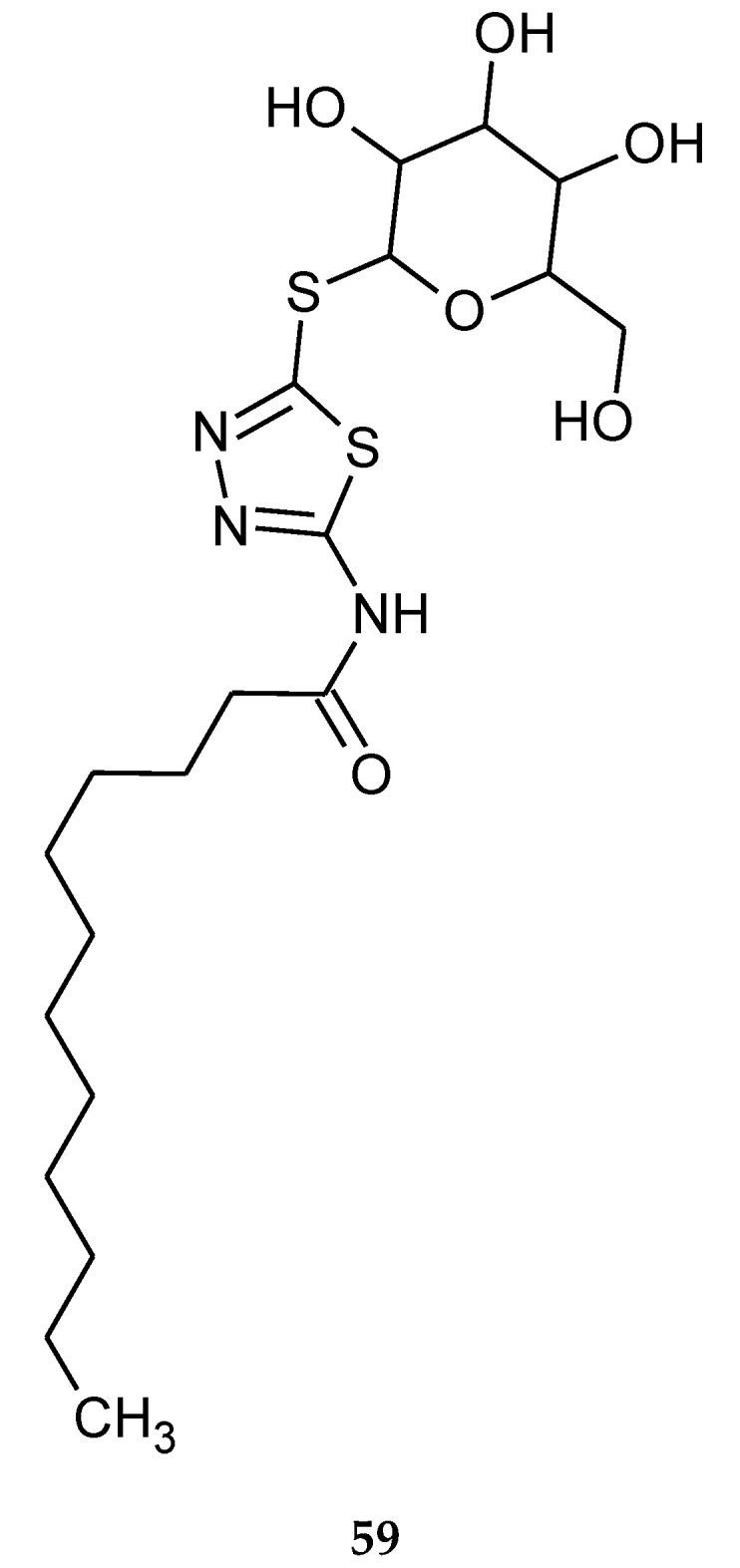
The structure of *N*-(5-(β-D-galactopyranosylthio)-1,3,4-thiadiazol-2-yl)dodecanamide (**59**).

1,3,4-Thiadiazole thioglycosides were also obtained by Vudhigiri et al. in 2017. The synthesized compounds were evaluated for their anticancer activity against four cancer cell lines and one normal cell line such as human ovarian cancer (SKOV3), cervical cancer (HeLa), breast cancer (MDAMB-231), human prostate cancer (DU145) and normal Chinese hamster ovary cancer (CHO-K1) cell line. New derivatives have shown moderate-to-promising activity during in vitro tests, with the IC_50_ values ranging from 8.7 to 97.5 µM. The most active compound (**59**) is presented in Figure 30. Acylated compounds were less active than compounds with a free hydroxyl group [39].

**Figure 31:**
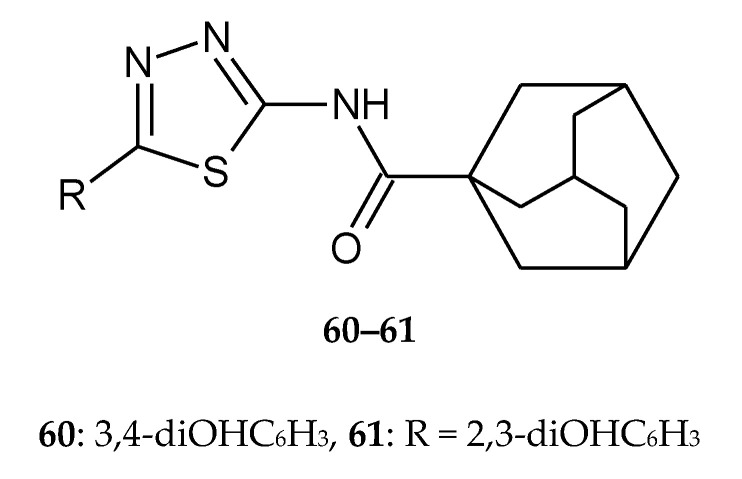
The structures of *N*-(5-(3,4-dihydroxyphenyl)-1,3,4-thiadiazol-2-yl)adamantane-1-carboxamide (**60**) and *N*-(5-(2,3-dihydroxyphenyl)-1,3,4-thiadiazol-2-yl)adamantane-1-carboxamide (**61**).

In the same year (2017), Jakovljević et al. reported synthesis, antioxidant and antiproliferative activities of 1,3,4-thiadiazoles derived from phenolic acids. The authors synthesized two series of 1,3,4-thiadia-zole-2-amides starting from 2,3-dihydroxybenzoic and 3,4-dihydroxybenzoic acid. Anticancer activity was evaluated against human lung cancer A549 and promyelocytic leukemia HL-60 and HeLa cell lines. The compounds prepared from 2,3-dihydroxybenzoic acid exhibited better antiproliferative activity. The most of compounds have shown good anticancer activity during in vitro tests and have not shown toxic activity against normal MRC-5 fibroblasts cell line. The study of the mechanisms of the antiproliferative action of the most active derivatives revealed that these compounds caused a significant increase in HL-60 cells within the subG1cell cycle phase. The induction of cell death in HL-60 cells was probably dependent on activation of caspase-3 and caspase-8. Two compounds (**60** and **61)** (Figure 31) were the most effective in inhibition of angiogenesis in vitro [40].

**Figure 32:**
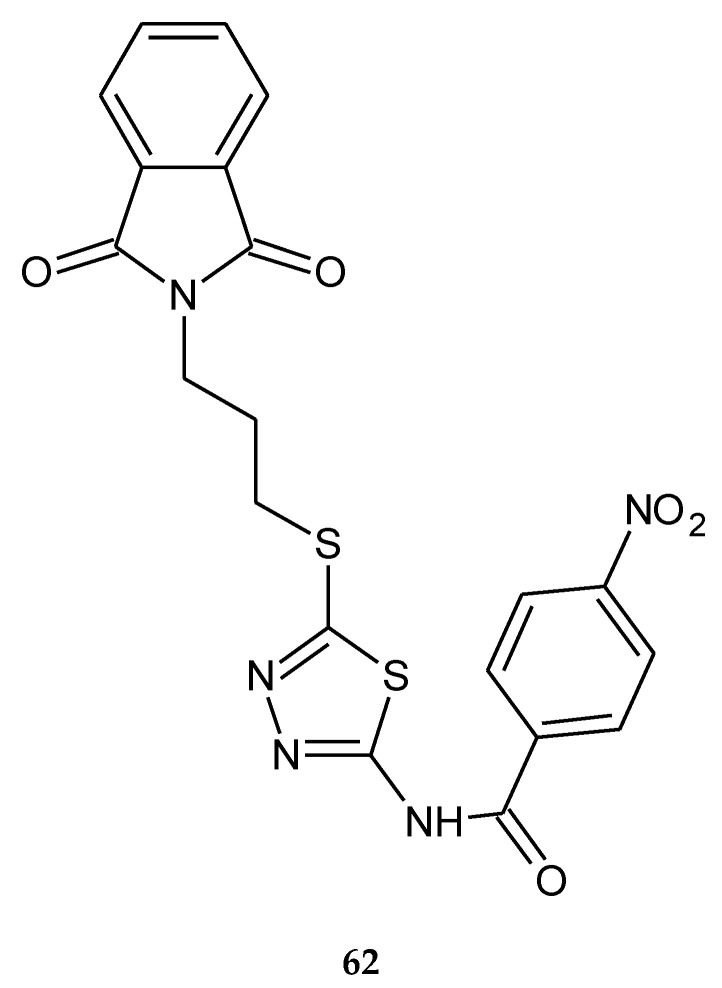
The structure of *N*-(5-{[3-(1,3-dioxo-1,3-dihydro-2*H*-isoindol-2-yl)propyl]sulfanyl}-1,2,4-thiadiazol-2-yl)-4-nitrobenzamide (**62**) (Table S1).

Rezaei and co-workers described synthesis and biological investigations of 1,3,4-thiadiazole-linked phthalimide derivatives (2017). The compounds were evaluated for their anticancer activity against human colon cancer HT-29 and human breast cancer MCF-7 cell lines, by MTT assay; the compound containing a 4-nitrobenzoyl moiety (**62)** (Figure 32) has shown the best anticancer activity, with IC_50_ = 23.83 and 27.21 μM, respectively [41].

**Figure 33:**
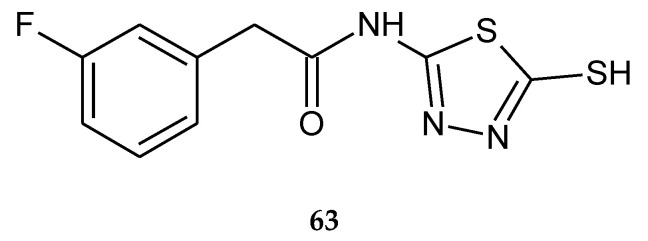
The structure of 2-(3-fluorophenyl)-*N*-(5-sulfanyl-1,3,4-thiadiazol-2-yl)acetamide (**63**) (Table S1).

In 2017, Mohammadi-Farani et al. reported cytotoxicity- and apoptosis-inducing effects of a series of *N*-(5-mercapto-1,3,4-thiadiazol-2-yl)-2-phenylacetamide derivatives. The anticancer activity was evaluated by an in vitro assay performed on three human cancer cell lines: prostate cancer (PC-3), breast cancer (MCF-7) and colon cancer (HT-29). The compound with 3-fluorophenyl substituent (**63**) (Figure 33) has shown the best activity against PC-3, with IC_50_ value 64.46 µM and against HT-29, with IC_50_ value 33.67 µM. Most of the analyzed derivatives showed a significant increase in activity of caspases 3, 8 and 9, in PC-3 and HT-29 cell lines compared to doxorubicin [42].

**Figure 34:**
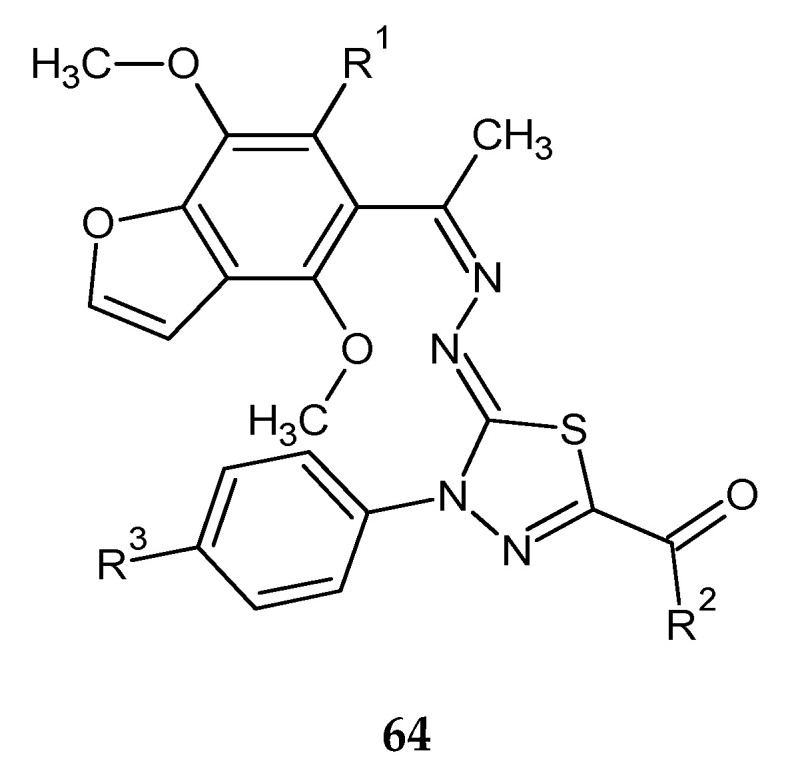
The structure of thiadiazole–benzofuran hybrids (**64**).

**Figure 35:**
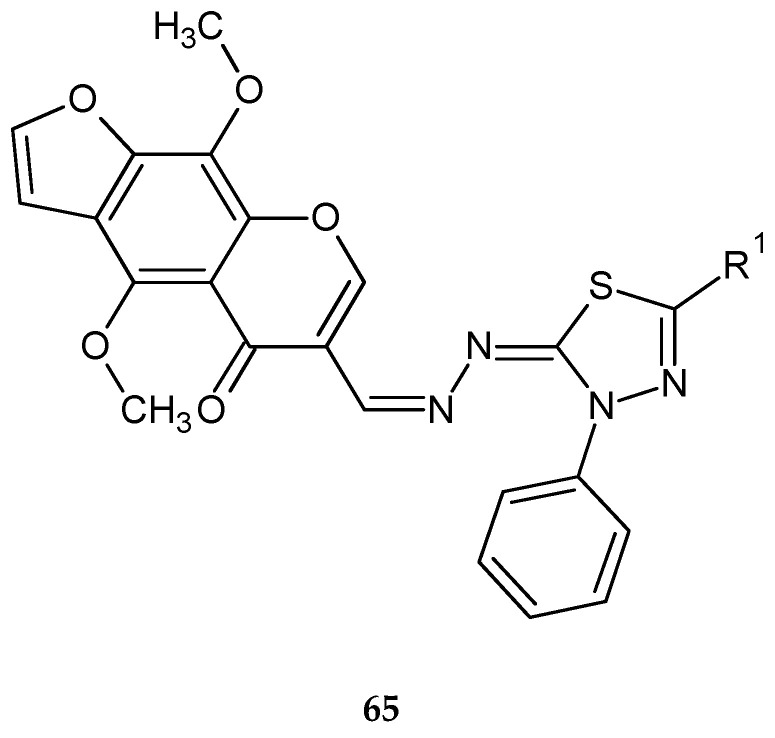
The structure of thiadiazole–furochromene hybrids (**65**).

Synthesis and biological evaluation of some novel thiadiazole–benzofurane (**64**) (Figure 34) and thiadiazole–furochromene hybrids (**65**) (Figure 35) were reported by Abdelhamid et al. (2018). Ten new compounds were evaluated for their anticancer activity against the human breast carcinoma (MCF-7) cell lines in comparison with reference doxorubicin using MTT assay. Some compounds demonstrated promising anticancer activity [43].

**Figure 36:**
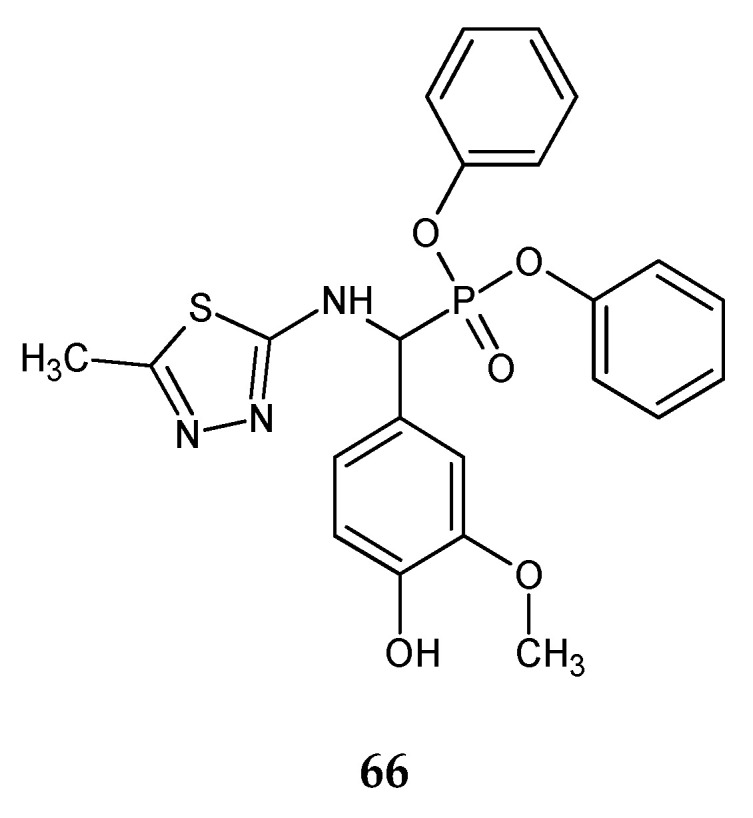
The structure of diphenyl(4-hydroxy-3-methoxyphenyl)(5-methyl-1,3,4-thiadiazol-2-ylamino)methylphosphonate (**66**).

In 2018, Azaam and co-workers reported anticancer activity of α-aminophosphonates containing a 1,3,4-thiadiazole moiety. The cytotoxic effects of the compounds on the human hepatocellular carcinoma (HepG2) and breast adenocarcinoma (MCF-7) cell lines were evaluated using MTT assay. The compound with a 4-hydroxy-3-metoxyphenyl substituent (**66**) (Figure 36) has shown the best anticancer activity during in vitro tests, with IC_50_ 18.17 µg/mL against HepG2 and 22.12 µg/mL against MCF-7 [44].

**Figure 37:**
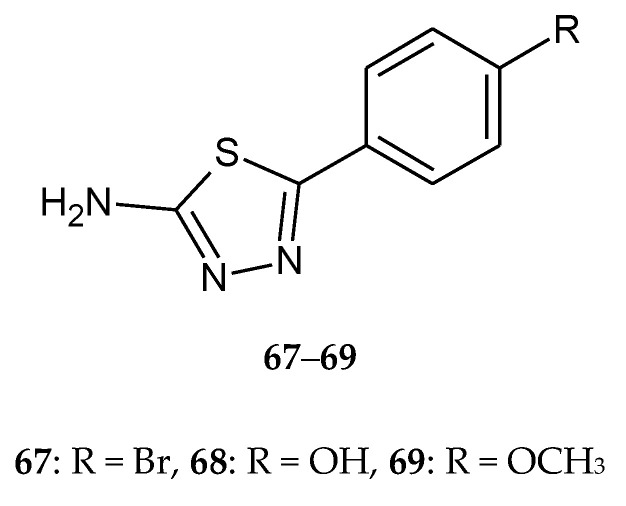
The structures of 2-amine-5-(4-R-phenyl)-1,3,4-thiadiazole (**67**–**69**).

Upadhyay et al. (2017) described synthesis, antimicrobial and anticancer activities of 5-(4-substituted-phenyl)-1,3,4-thiadiazole-2-amines. Anticancer study of synthesized compounds was carried out against human breast cell line (MCF-7) using SRB assay. Compounds with bromo-, hydroxyl- and methoxy- substituents (**67**–**69**) (Figure 37) have shown moderate-to-good anticancer activity with GI_50_ values ranging from 24.0 to 46.8 µg/mL [45].

**Figure 38:**
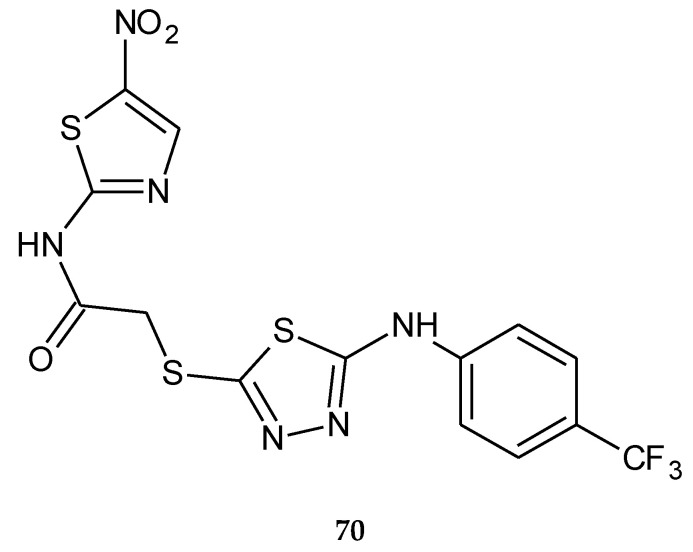
The structure of *N*-(5-nitrothiazol-2-yl)-2-((5-((4-(trifluoromethyl)phenyl)amino)-1,3,4-thiadiazol-2-yl)thio)acetamide (**70**).

In 2018, Altinop and co-workers reported anticancer activity in a series of 1,3,4-thiadiazole derivatives. The compounds were evaluated for their anticancer activity against human cancer cell lines. *N*-(5-Nitrothiazol-2-yl)-2-((5-((4-(trifluoromethyl)phenyl)amino)-1,3,4-thiadiazol-2-yl)thio)acetamide (**70**) (Figure 38) has shown selective anticancer activity during in vitro tests. This compound inhibited the Abl protein kinase with an IC_50_ value of 7.4 µM and showed selective activity against the Bcr-Abl-positive K562 cell line (myelogenous leukemia cell line**)**. Molecular studies showed that this compound forms hydrogen bonding between the nitro group and Met318 in addition to Tyr253-thiazole, and Thr315-thiazole interactions and the trifluoromethylphenyl group are enclosed by a hydrophobic region of the pocket with Ile293, Leu298, and Leu354. The compound without the nitro group does not show interaction with Met318, Tyr253, or Thr315 residues and does not inhibit the K562 cell line, suggesting significant role of the nitro group in activity against Abl protein kinase [46].

**Figure 39:**
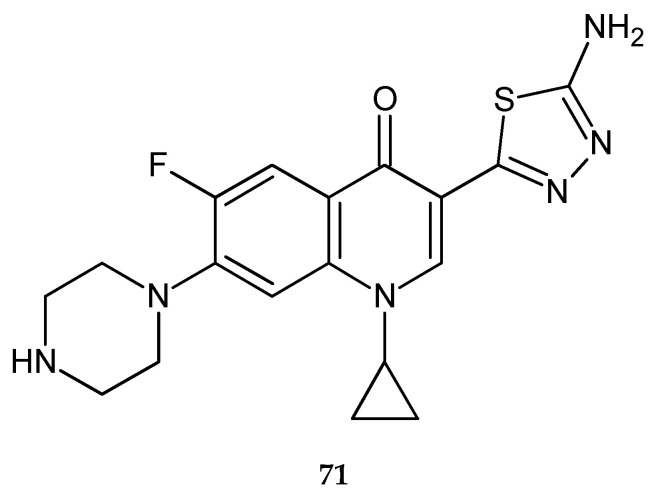
The structure of {(3-(5-amino-1,3,4-thiadiazol-2-yl)-1-cyclopropyl-6-fluoro-7-(piperazin-1-yl)quinoline-4(1*H*)-one)} (**71**).

**Figure 40:**
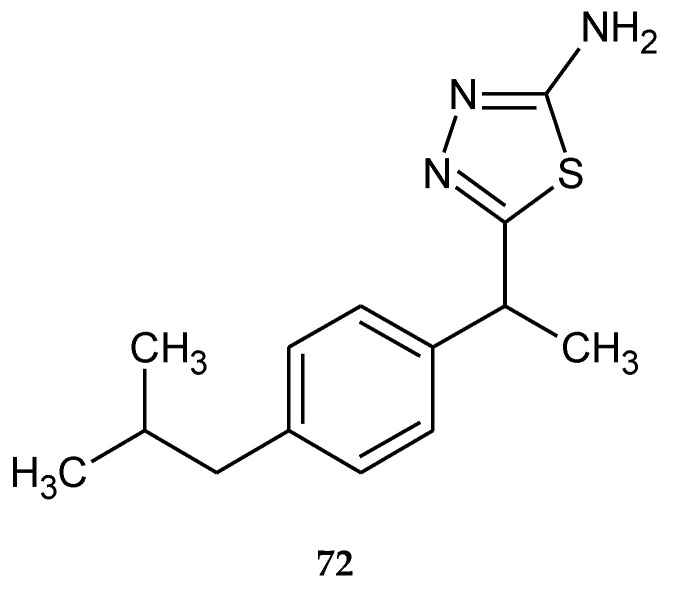
The structure of 2-amino-5-(1-(4-isobutylphenyl)ethyl)-1,3,4-thiadiazole (**72**).

Synthesis, theoretical, spectroscopic and electrochemical DNA binding investigations of 1,3,4-thiadiazole hybrids with ibuprofen and ciprofloxacin were reported by Farooqi et al. (2018). The cytotoxic effects of new synthesized compounds were evaluated by using human hepatocellular carcinoma cell lines (Huh-7) in MTT assay. All compounds have shown good anticancer activity during in vitro tests. The best activity has been shown by ciprofloxacin derivative (**71**) (Figure 39), with IC_50_ increase of 25.75 μM. Ibuprofens derivative (**72**) (Figure 40) was less active. Theoretical and experimental results suggested spontaneous and significant intercalative binding of the compounds with DNA [47].

**Figure 41:**
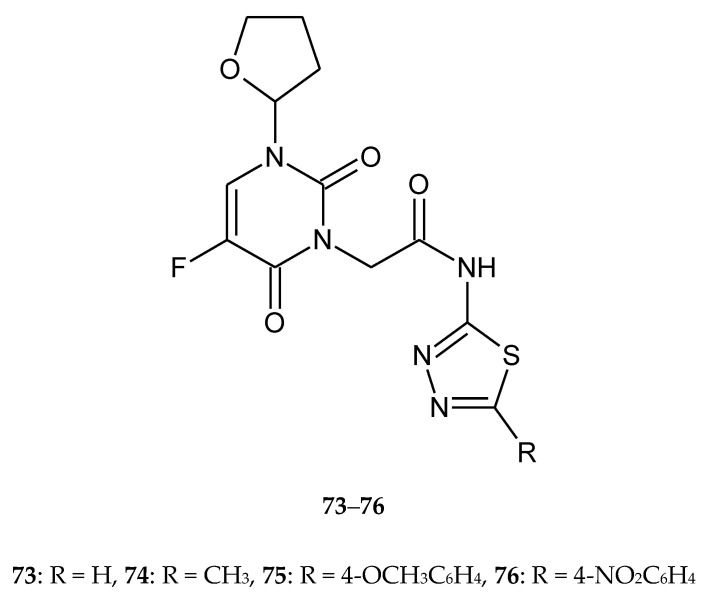
The structures of 5-fluorouracil derivatives (**73**–**76**).

Liu and co-workers (2017) reported biological activity of new tegafur (prodrug of 5-fluorouracil) derivatives containing a 1,3,4-thiadiazole moiety **73**–**76** (Figure 41). The compounds were evaluated for their anticancer activity against gastric cancer (HGC27), human hepatocarcinoma (SMMC7721), colon adenocarcinoma (HCT15) and human bronchial epithelial (Beas-2B) cell lines. All compounds have shown good anticancer activity during in vitro tests. Four tegafur derivatives showed IC_50_ values in the range of 0.16~100 μg/mL. The new derivatives were less toxic for normal cell lines [48].

**Figure 42:**
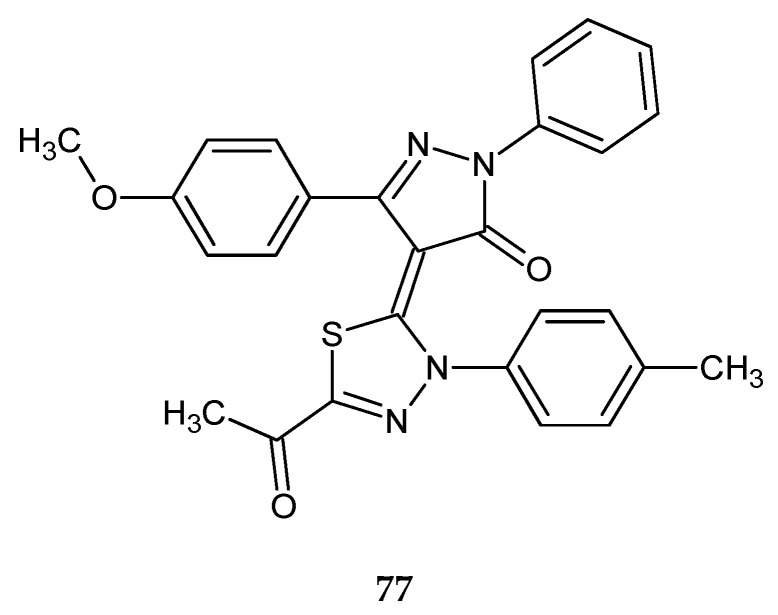
The structure of 4-(5-acetyl-3-*p*-tolyl-1,3,4-thiadiazol-2(3*H*)-ylidene)-3-(4-methoxyphenyl)-1-phenyl-1*H*-pyrazol-5(4*H*)-one (**77**).

In 2018, Fathy et al. reported anticancer activity of a series of new pyrazoline-based 1,3-oxathioles and 1,3,4-thiadiazoles. The compounds were evaluated for their anticancer activity against human liver cancer (HepG-2) and human breast cancer (MCF-7) cell lines, by MTT assay. 1,3-Oxathiazole derivatives were the most potent compounds against HepG-2 and MCF-7 cancer cells, whereas among thiadiazole derivatives the most active was compound **77** (Figure 42). It showed significant anticancer activity against HepG-2 and MCF-7 cancer cells with IC_50_ = 84.9 ± 5.9 µM and 63.2 ± 2.9 µM, respectively [49].

**Figure 43:**
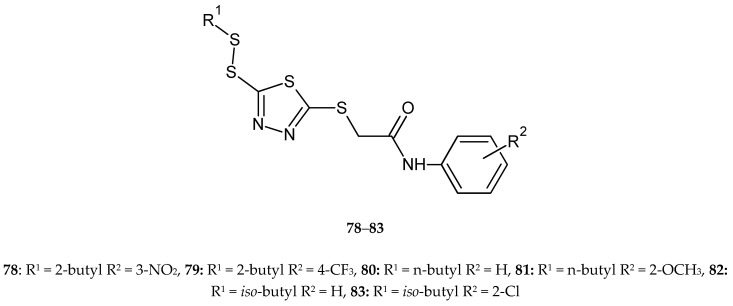
The structures of 2-(R^1^-disulfanyl)-5-[(R^2^-phenylcarbamoyl)-methylthio]-1,3,4-thiadiazole (**78**–**83**).

Zhang and co-workers (2017) reported synthesis and anticancer activity of new disulfides containing a 1,3,4-thiadiazole moiety. The compounds were evaluated for their anticancer activity against lung cancer (A549), HeLa, hepatocarcinoma (SMMC-7721) and mouse fibroblasts (L929) cell lines by CCK-8 assay. Some of the new compounds inhibited the proliferation better than fluorouracil. Compound **78** was the most active against A549 cells with IC_50_ value of 3.62 μM. Compounds **79**–**83** displayed significantly antiproliferative activities against HeLa cells with IC_50_ values of 3.88, 3.76, 3.59, 3.38 and 3.12 μM, respectively. Compounds **78**, **80** and **82** demonstrated high antiproliferative activities against SMMC-7721 cells with IC_50_ values of 2.54, 2.69 and 2.31 μM (Figure 43). All the tested compounds showed a weak cytotoxic effect against the normal cell line L929 [50].

**Figure 44:**
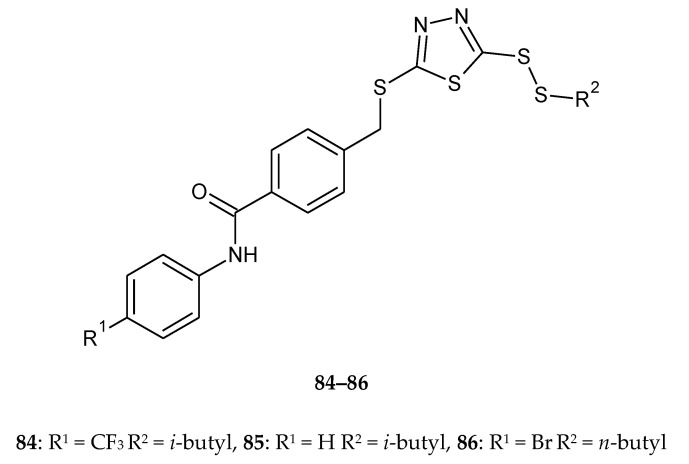
The structures of 2-(*iso/n*-butyldisulfanyl)-5-[4-(4-R-phenylcarbamoyl)-benzylthio]-1,3,4-thiadiazole (**84**–**86**) (Table S1).

The synthesis and anticancer activity of new disulfides containing a 1,3,4-thiadiazole moiety were reported by Liu et al. in 2018. The compounds were evaluated for their anticancer activity against lung cancer (A549), HeLa, hepatocarcinoma (SMMC-7721) and mouse fibroblasts (L929) cell lines by CCK-8 assay. Some of the compounds showed better antiproliferative activities than reference drug- 5-fluorouracil. The compounds **84**–**86** (Figure 44) demonstrated the best anticancer activity against A549 cell lines, with values of IC_50_ ranging from 2.12 to 4.58 μM, while value of IC_50_ for reference drug was 8.13 μM. All the tested compounds displayed a weak cytotoxic effect against the normal cell line L929 [51].

**Figure 45:**
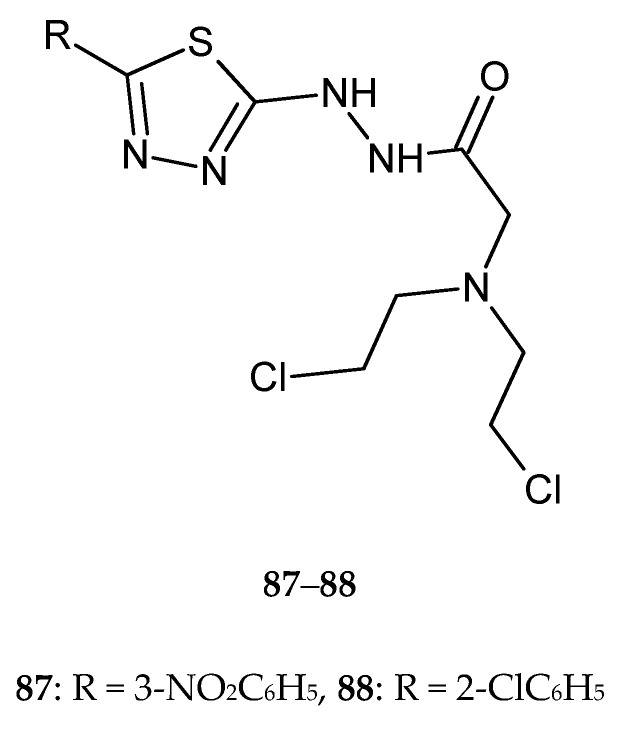
The structures of 2-[bis(2-chloroethyl)amino]-*N*-[5-(3-nitrophenyl)-1,3,4-thiadiazol-2-yl)acetohydrazide (**87**) and 2-[bis(2-chloroethyl)amino]-*N*-[5-(2-chlorophenyl)-1,3,4-thiadiazol-2-yl)acetohydrazide (**88**).

In 2018, Gowramma and co-workers reported anticancer activity of the series of new 1,3,4-thiadiazole derivatives. The compounds were evaluated for their anticancer activity against human liver cancer (Hep-2) cell line by CCK-8 assay. Compounds with 3-nitrophenyl and 2-chlorophenyl substituents **87**–**88** (Figure 45) demonstrated substantial antitumor activity [52].

**Figure 46:**
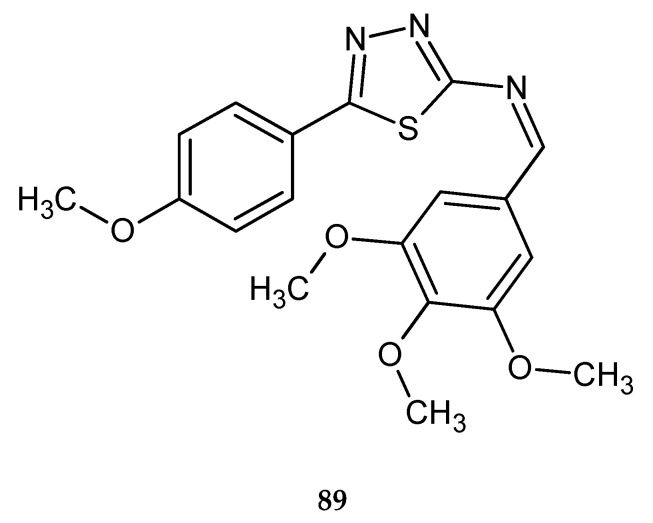
The structure of [5-(4-methoxyphenyl)-1,3,4-thiadiazol-2-yl]-(3,4,5-trimethoxybenzylidene)amine (**89**).

New utilities for colon cancer treatment for 2-amino-5-substituted-1,3,4-thiadiazole was described by Raj and co-workers (2018). The authors used the molecular dynamic simulation to find out the most potent lead compounds. Later, SRB assay using human colon cancer (HT-29) cells and ELISA assays were performed to explore activity and molecular targets of new derivatives. In vitro tests have confirmed anticancer activity of the tested compounds. One compound **89** (Figure 46) has shown the best antitumor activity with GI_50_ < 0.1 µM [53].

**Figure 47:**
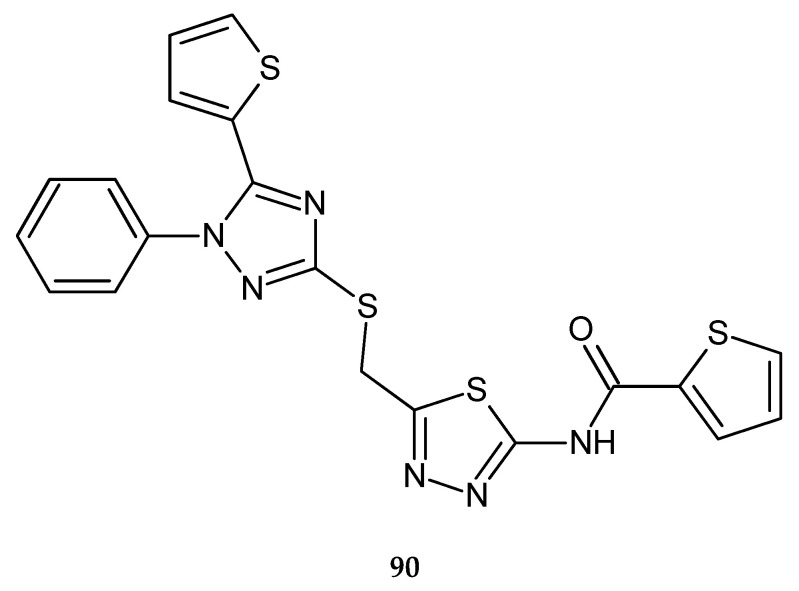
The structure of *N*-[5-({[1-phenyl-5-(thiophen-2-yl)-1*H*-1,2,4-triazol-3-yl]sulfanyl}methyl)-1,3,4-thiadiazol-2-yl}thiophene-2-carboxyamide (**90**).

In 2018, Nassar et al. reported anticancer activity of 1,2,4-triazole, 1,3,4-oxadiazole and 1,3,4-thiadiazole (**90**) (Figure 47) derivatives. The compounds were evaluated for their anticancer activity against hepatocellular carcinoma HePG-2 and breast cancer MCF-7 cell lines by MTT assay. The anticancer activities of the compounds were good [54].

**Figure 48:**
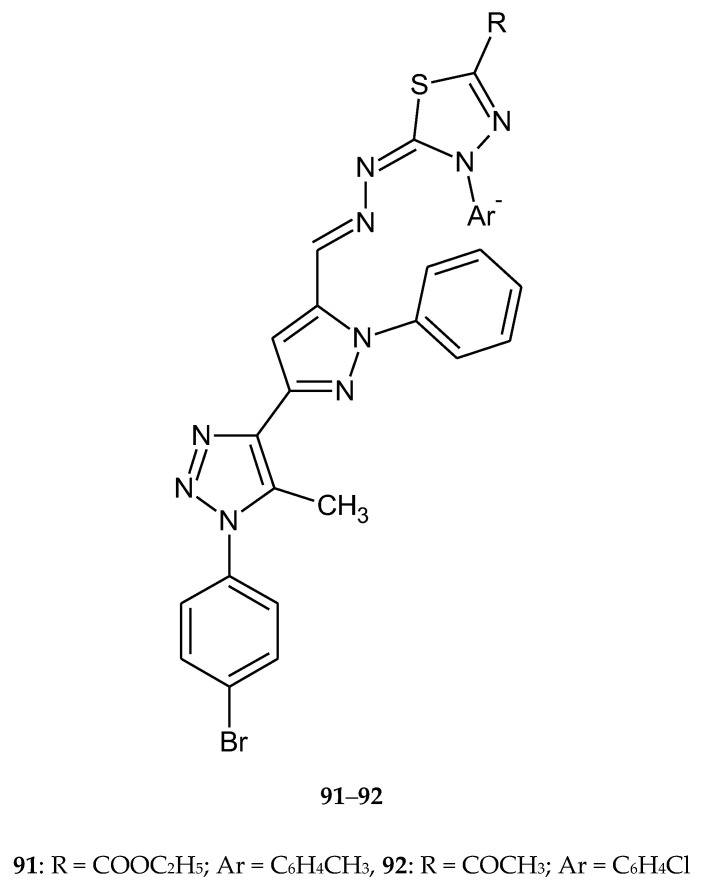
The structure of most active compounds: ethyl 5-((-(3-(1-(4-bromophenyl)-5-methyl-1*H*-1,2,3-triazol-4-yl)-1-phenyl-1*H*-pyrazol-4-yl)methylene)hydrazono)-4-(4-tolyl)-4,5-dihydro-1,3,4-thiadiazole-2-carboxylate (**91**) and 1-(5-((-(3-(1-(4-bromophenyl)-5-methyl-1*H*-1,2,3-triazol-4-yl)-1-phenyl-1*H*-pyrazol-4-yl)methylene)hydrazono)-4-(4-chlorophenyl)-4,5-dihydro-1,3,4-thiadiazol-2-yl)ethan-1-one (**92**).

In 2019, Rashdan et al. described synthesis and anticancer activity new 1,3,4-thiadiazole derivatives. The synthesis was performed under solvent free conditions and newly compounds were screened for anticancer activity towards human breast carcinoma (MCF-7) and human lung carcinoma (A-549). Most of the tested compounds showed remarkable anti-breast cancer activity. Compounds **91** and **92** (Figure 48) had good effects on cancer cells with safety effects on normal cells. They induce apoptosis inside the cells and increase DNA fragmentation [55].

**Figure 49:**
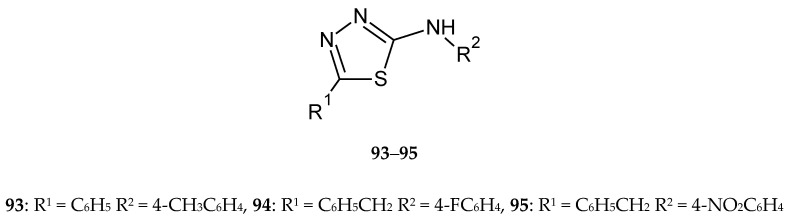
The structure of most active 2-amino-5-phenyl-substituted 1,3,4-thiadiazole (**93**–**95**).

Chandra et al. (2019) synthesized and screened a series of novel 5-phenyl-substituted 1,3,4-thiadiazole-2-amines for their antitumor and antitubercular activities. Some of the synthesized compounds (**93**–**95**) (Figure 49) showed significant antitumor activities against breast cancer (MDA MB-231). Among them, *N*-benzyl-5-(4-fluorophenyl)-, *N*-benzyl-5-(4-nitrophenyl)-, and 5-phenyl-*N*-(4-tolyl)-1,3,4-thiadiazole-2-amines demonstrated higher inhibitory activities than cisplatin used as reference drug. The SAR analysis in this group showed that an aromatic ring and electron-withdrawing substituents promote anticancer activity [56].

**Figure 50:**
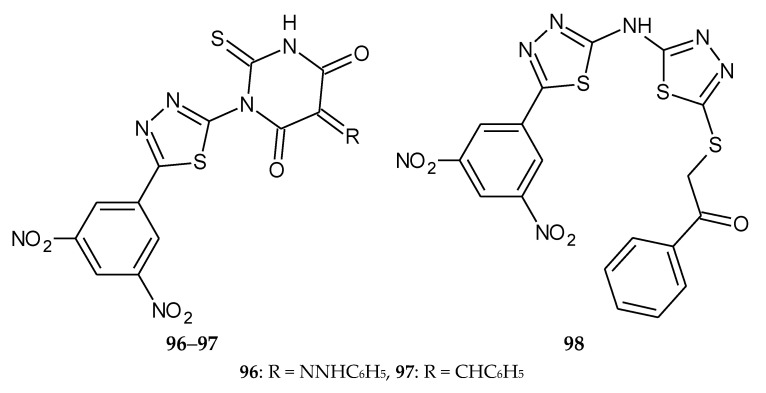
The structures of 2-((5-((5-(3,5-dinitrophenyl)-1,3,4-thiadiazol-2-yl)amino)-1,3,4-thiadiazol-2-yl)thio)-1-phenylethan-1-one (**96**), 1-[5-(3,5-dinitrophenyl)-1,3,4-thiadiazol-2-yl]-3-phenyl-5-(2-phenylhydrazono)-2-thioxo-dihydro-pyrimidine-4,6(1*H*,5*H*)-dione (**97**) and 5-benzylidene-1-[5-(3,5-dinitrophenyl)-1,3,4-thiadiazol-2-yl]-3-phenyl-2-thioxo-dihydro-pyrimidine-4,6(1*H*,5*H*)-dione (**98**).

**Figure 51:**
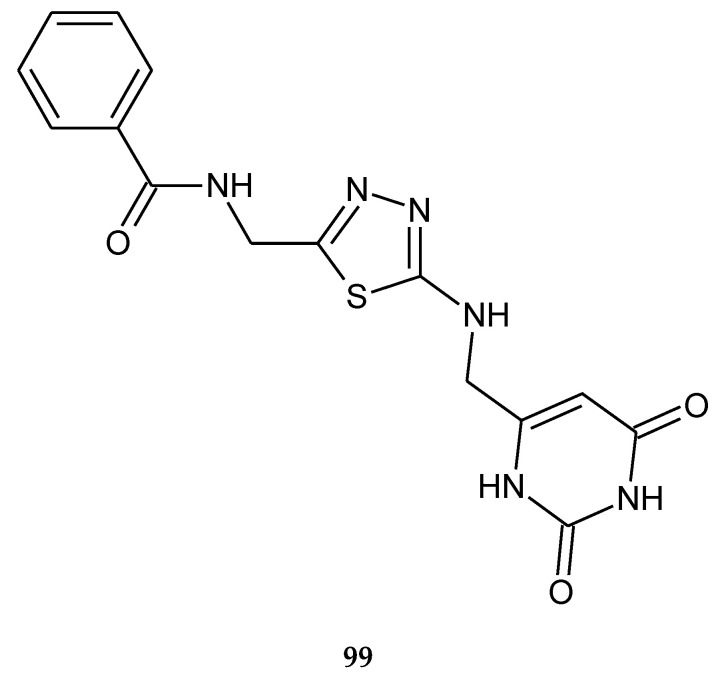
The structure of *N*-((5-(((2,6-dioxo-1,2,3,6-tetrahydropyrimidin-4-yl)methyl)amino)-1,3,4-thiadiazol-2-yl)methyl)benzamide (**99**).

**Figure 52:**
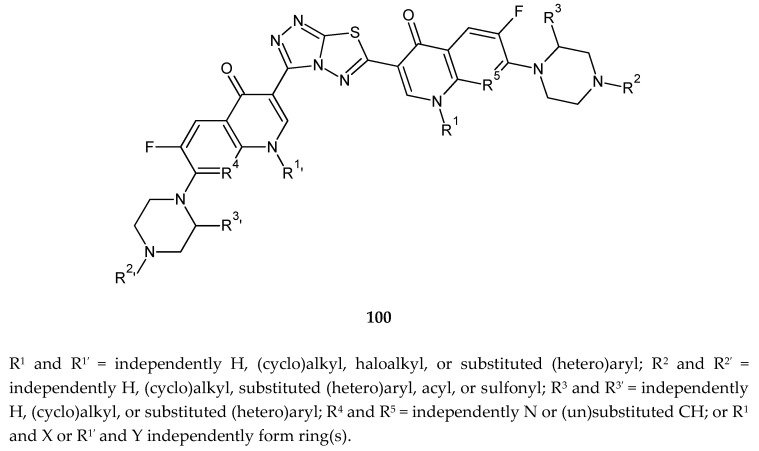
The structure of new derivatives of 1,2,4-triazolo[3,4-b][1,3,4]thiadiazole (**100**).

El-Naggar et al., in 2019, published a synthesis of a new series of 5-(3,5-dinitrophenyl)-1,3,4-thiadiazole derivatives and a biological evaluation for their in vitro antimicrobial, antitumor, and DHFR (Dihydrofolate reductase) inhibition activity. Three compounds, **96**–**98** (Figure 50), **76** (Figure 51) and **77** (Figure 52), exhibited antitumor activity against four human cancer cell lines, CCRF-CEM leukemia, HCT-15 colon, PC-3 prostate, and UACC-257 melanoma. Doxorubicin was used as a reference drug. The compounds were characterized by selectivity of action against tumor cells [57].

Mahapatra et al. (2019) described anticancer activity of *N*-((5-(((2,6-dioxo-1,2,3,6-tetrahydropyrimidin-4-yl)methyl)amino)-1,3,4-thiadiazol-2-yl)methyl)benzamide (**99**) (Figure 51) against MCF-7 breast cancer cell line. The IC_50_ value was established in comparison with capecitabine—the reference drug. The activity of the compound was weaker than this drug [58].

## 3. 1,3,4-Thiadiazole Condensed with Other Heterocyclic Rings

### 3.1. Triazole–Thiadiazole Hydrids

Hu et al. (2010) reported anticancer and antimicrobial activity of a series of 1,2,4-triazolo[3,4-b][1,3,4]thiadiazole (**100**) (Figure 52). Triazolo–thiadiazole rings were connected with the fluoroquinolone system. The compounds have shown growth-inhibiting activities of tumor cell lines HL-60 (human leukemia) and DBA/2 with IC_50_ of 1.1, 0.25, and 0.15 μM [59].

**Figure 53:**
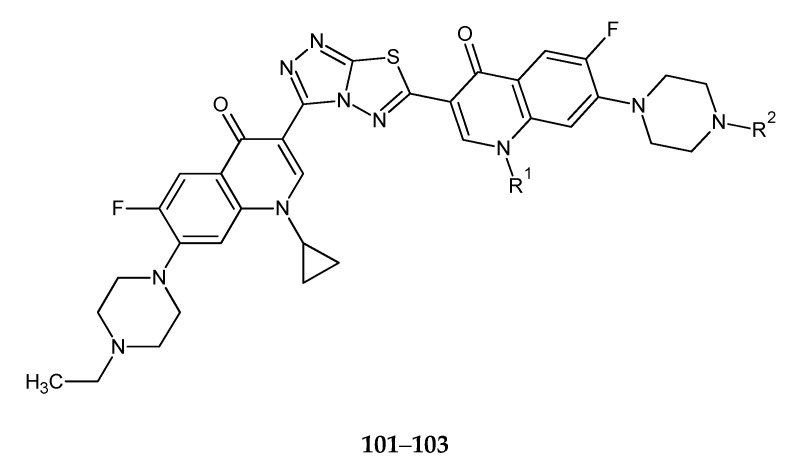
The structures of 3,6-bis-(cyclopyopyl-C3/C3-fluoroquinolone)substituents-triazolo[2,1-b][1,3,4]thiadiazoles (**101**–**103**).

The next work, published by the same authors, reported synthesis and anticancer evaluation of five C3/C3 fluoroquinolone dimers tethered with a fused heterocyclic ring with 1,3,4-thiadiazole groups (**101**–**103**) (Figure 53). The anticancer activity of the compounds was evaluated against L1210 (mouse lymphocytic leukemia) and CHO (Chinese hamster ovary) cell lines. Compounds with R^1^/R^2^ = cyclopropyl/H (**101**); cyclopropyl/CH_3_ (**102**); cyclopropyl/C_2_H_5_ (**103**) have shown the best activity with IC_50_ values of 0.20, 1.2 and 2.5 μmol/L [60].

**Figure 54:**
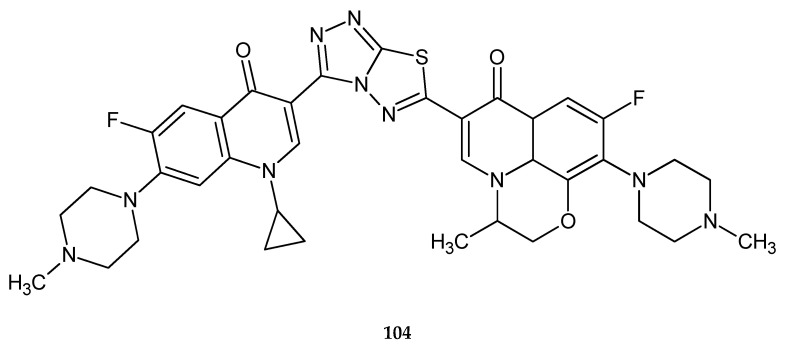
The structure of (S)-1,2,4-triazolo[3,4-b][1,3,4]thiadiazole-3-[1-cyclopropyl-6-fluoro-7-piperazin-1-yl-quinolin-4(1*H*)-one]-6-[1,8-(2,1-oxy-propyl)-6-fluoro-7-(4-methylpiperazin-1-yl)-quinolin-4(1*H*)-one] (**104**).

Continuing research on fluoroquinolone derivatives, Hu and co-workers (2011) reported synthesis and anticancer activity in a series of new compounds with 1,3,4-thiadiazole rings. The compounds were evaluated against L1210 (lymphocytic leukemia), CHO (Chinese hamster ovary) and HL60 (human leukemia) cell lines in the MTT assay. Some of the compounds exhibited good cytotoxicity against cell lines, with IC_50_ values of 1.5 μM (L1210), 0.12 μM (HL60) and 3.4 μM (CHO) for compounds with methyl substituent **104** (Figure 54) [61].

**Figure 55:**
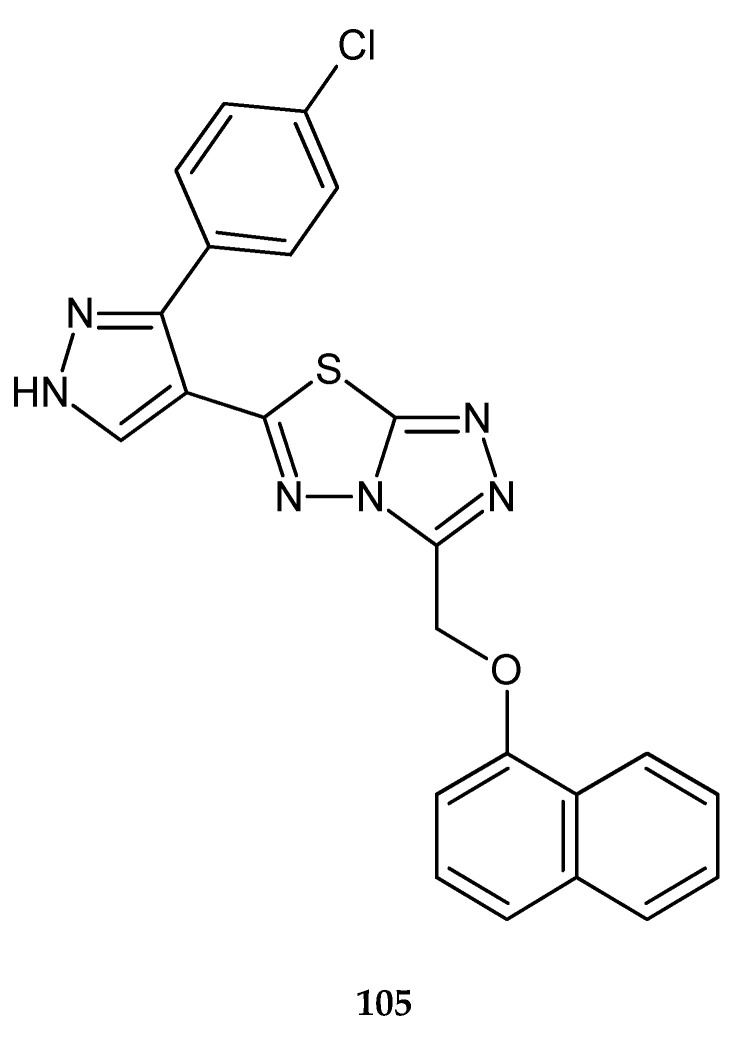
The structure of 6-[3-(4-chlorophenyl)-1*H*-pyrazol-4-yl]-3-[2-naphthyloxy)methyl] [1,2,4]triazolo[3,4-][1,3,4]thiadiazole (**105**).

Sunil and co-workers (2011) reported synthesis and anticancer evaluation of new compound: 6-[3-(4-chlorophenyl)-1H-pyrazol-4-yl]-3-[2-naphthyloxy)methyl][1,2,4]triazolo[3,4-b][1,3,4]thia-diazole **105** (Figure 55). It demonstrated growth-inhibiting activities with an IC_50_ value of 0.8 μg/mL for tumor cell line HepG2 (liver cancer) [62].

**Figure 56:**
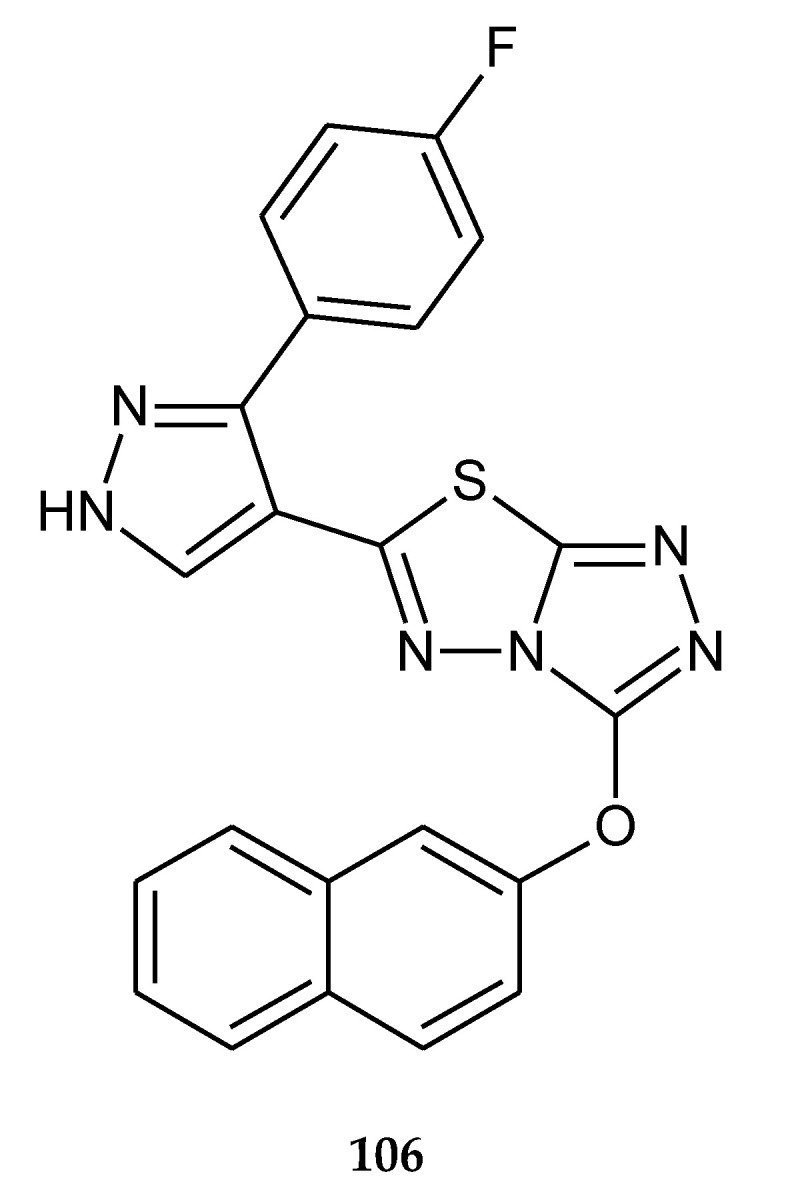
The structure of 6-[3-(4-fluorophenyl)-1*H*-pyrazol-4-yl]-3-[(2-naphthyloxy)methyl][1,2,4]triazolo[3,4-b][1,3,4]thiadiazole (**106**) (Table S2).

The same authors published results of anticancer investigations of 6-[3-(4-fluorophenyl)-1*H*-pyrazol-4-yl]-3-[(2-naphthyloxy)methyl][1,2,4]triazolo[3,4-b][1,3,4]thiadiazole (**106**) (Figure 56). The compounds characterized activity higher than doxorubicin against liver cancer cells HepG2. The authors indicated using the flow cytometric test that higher percentage of cells were in sub-G1 phase. Next, they observed in chromatin condensation studies by Hoechst staining indication that cells were in apoptosis [63].

**Figure 57:**
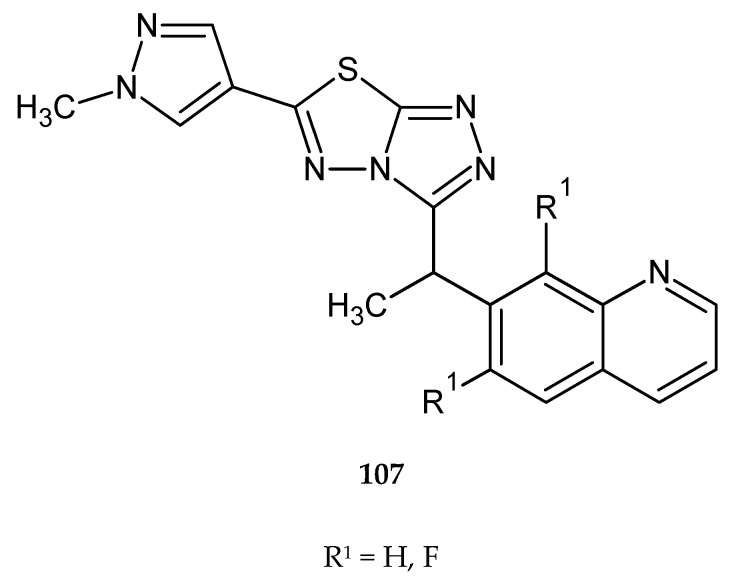
The structure of new [1,2,4]triazolo[3,4-b][1,3,4]thiadiazole derivatives (**107**).

In 2010, Lauffer and co-workers described synthesis and anticancer evaluation of two new compounds with 1,3,4-thiadiazole condensed rings (**107**) (Figure 57). The new complexes were active against c-Met protein kinase in gastric carcinoma cells Snu5. IC_50_ values in the test were <200 nM [64].

**Figure 58:**
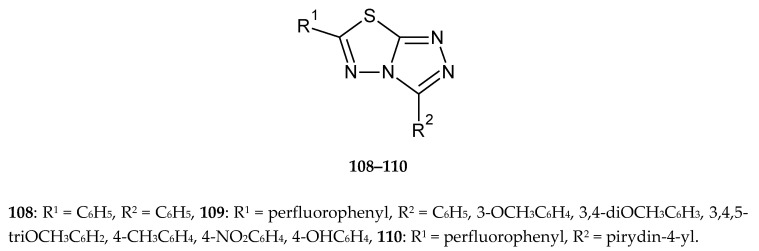
The structures of 3,6-diphenyl-[1,2,4]triazolo[3,4-b][1,3,4]thiadiazole (**108**), 6-(perfluorophenyl)-3-R-phenyl-[1,2,4]triazolo[3,4-b][1,3,4]thiadiazole (**109**) and 6-(perfluorophenyl)-3-(pyridin-4-yl)-[1,2,4]triazolo[3,4-b][1,3,4]thiadiazole (**110**).

The series of condensed fluorinated 3,6-diaryl-[1,2,4]triazolo[3,4-b][1,3,4]thiadiazoles was obtained by Chowrasia et al. in 2017. The compounds were evaluated for their anticancer activity against cancer cell lines MCF-7, SaOS-2 and K562. Some new derivatives have shown good cytotoxicity against investigated cells, with the best IC_50_ values 22.1, 19.0 and 15 μM against MCF7 (breast cancer), SaOS-2 (human osteosarcoma) and K562 (myelogenous leukemia), respectively e.g., compounds without substituents in phenyl ring in position 3 of triazole (**108**) (Figure 58). The introduction of fluorine atoms (**109**) causes an increase in activity while replacing the phenyl ring with a pyridine does not change the activity (**110**) [65].

**Figure 59:**
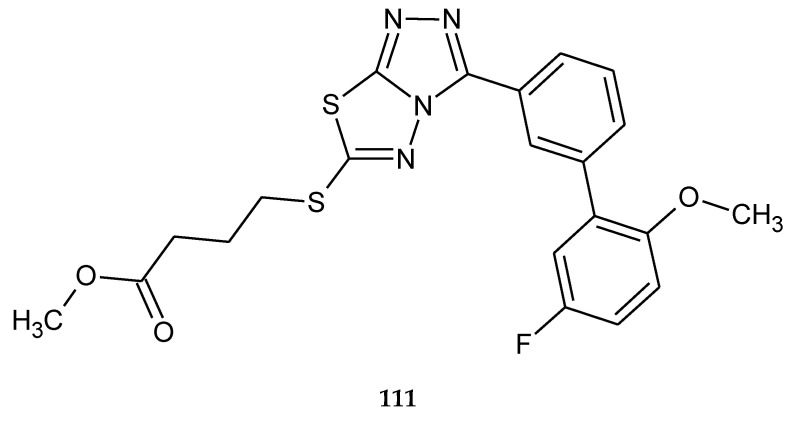
The structure of 6-((4-fluorobutyl)sulfanyl)-3-(5’-fluoro-2’-methoxy-(1,1’-biphenyl)-3-yl)-1,2,4-triazolo[3,4-b][1,3,4]thiadiazole (**111**).

Ramaprasad and co-workers obtained series of new condensed triazolo–thiadiazole analogues using microwave radiation. The compounds were evaluated for their anticancer activity against HT29 (colon cancer) cell lines by MTT assay. The best IC_50_ value was 12 μM for 6-((4-fluorobutyl)sulfanyl)-3-(5’-fluoro-2’-methoxy-(1,1’-biphenyl)-3-yl)-1,2,4-triazolo[3,4-b][1,3,4]thiadiazole (**111**) (Figure 59) [66].

**Figure 60:**
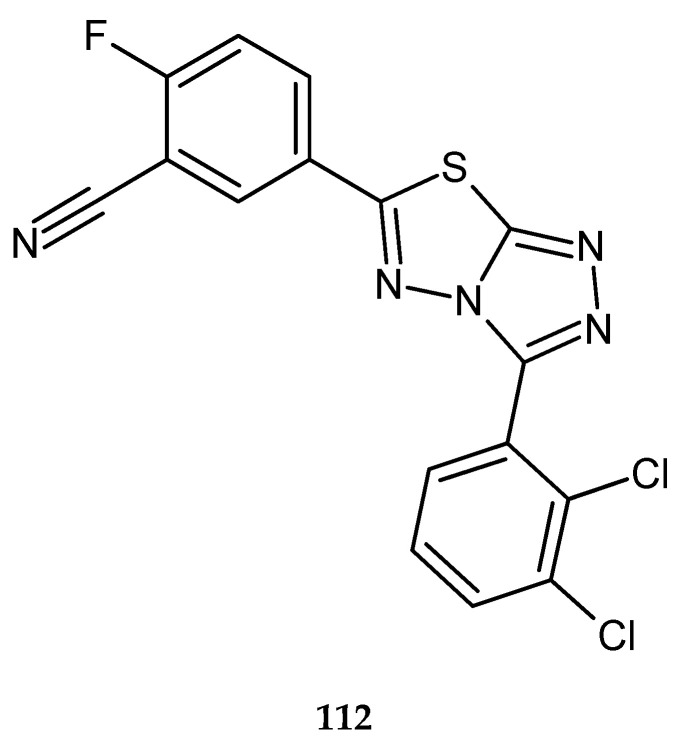
The structure of 5-(3-(2,3-dichlorophenyl)-[1,2,4]triazolo[3,4-*b*][1,3,4]thiadiazol-6-yl)flurobenzonitrile (DTTF) (**112**).

Inhibition of tumor cell invasion and increase in the apoptotic effect of THFα (tumor necrosis factor α) by abrogating NF-κB activation cascade by a series of condensed-bicyclic 4,6-substituted 1,2,4-triazolo-1,3,4-thiadiazoles were described by Ningegowda and co-workers in 2017. The compounds were evaluated for their anticancer activity against SiHa (human cervical cancer), Caski (human cervical carcinoma), and HeLa (cervical cancer) cell lines, by MTT assay. 5-(3-(2,3-Dichlorophenyl)-[1,2,4]triazolo[3,4-*b*][1,3,4]thiadiazol-6-yl)flurobenzonitrile (DTTF) **112** (Figure 60) has shown the best activity against SiHa cancer cells, with an IC_50_ value of 15.6 µM [67].

**Figure 61:**
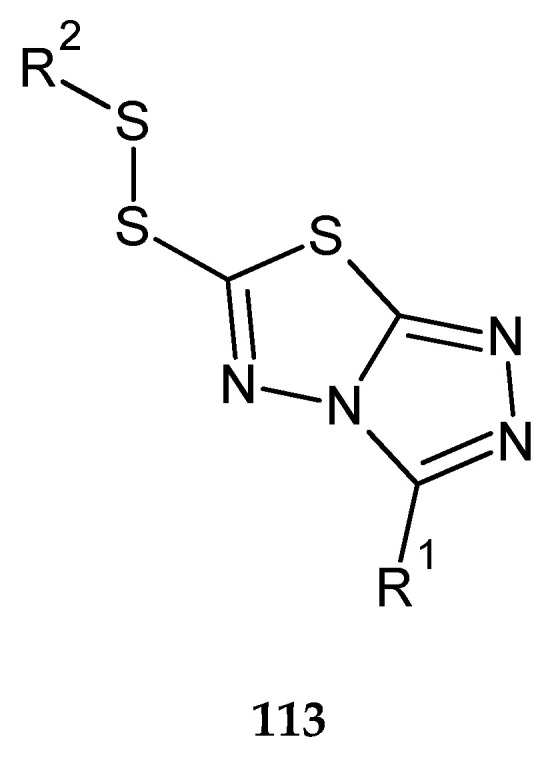
The structure of 3-R^1^-6-(R^2^-disulfanyl)[1,2,4]triazolo[3,4-b][1,3,4]thiadiazole (**113**) (Table S2).

In the same year (2017), Chen and co-workers patented anticancer activity of 1,2,4-triazolo[3,4-b]-1,3,4-thiadiazole. The compounds were evaluated for their cytotoxicity against HeLa (cervical cancer), SMMC-7721 (hepatocarcinoma) and A549 (human lung carcinoma) cell lines. The compounds with substituents R^1^ = C_6_H_5_, 4-CH_3_OC_6_H_5_, 4-ClC_6_H_4_, 4-FC_6_H_4_, or 3,4,5-triOCH_3_C_6_H_2_; R^2^ = C_4_H_9_, iso-C_4_H_9_, 2-C_4_H_9_ **113** (Figure 61) have shown good anticancer activity during in vitro tests [68].

**Figure 62:**
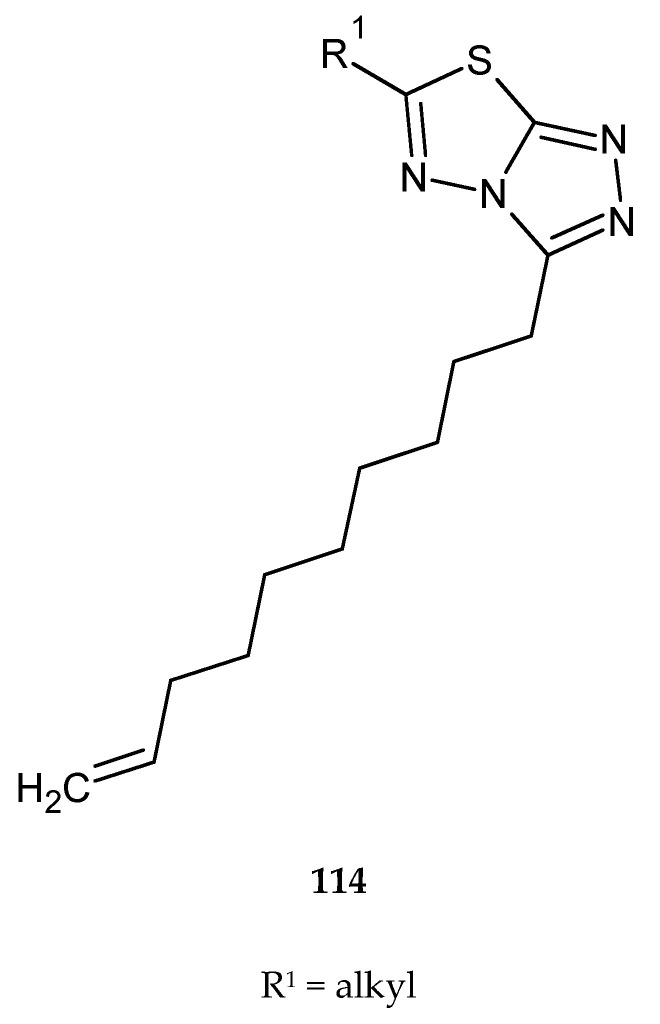
The structure of 3,6-dialkylsubstituted-[1,2,4]triazolo[3,4-b][1,3,4]thiadiazoles (**114**).

Synthesis and evaluation of anticancer activity of 3,6-dialkylsubstituted-[1,2,4]triazolo[3,4-b][1,3,4]thiadiazoles (**114**) (Figure 62) were reported by Venepally and co-workers in 2018. The compounds were tested for their anticancer activity against MCF-7 (breast cancer), HeLa (cervical cancer), B16-F10 (melanoma) and SKOV3 (ovarian cancer) cell lines, by MTT assay. Most of the tested derivatives have shown promising anticancer activity, with IC_50_ values between 13.67 and 18.62 μM for B16-F10 cells. Hexyl-, decyl-, undecenyl-, lauryl-, myristyl-, palmityl-, stearyl- and oleyl-based derivatives exhibited significant activities against SKOV3 and MCF-7 cell lines, whereas they were not toxic for the normal cell line [69].

**Figure 63:**
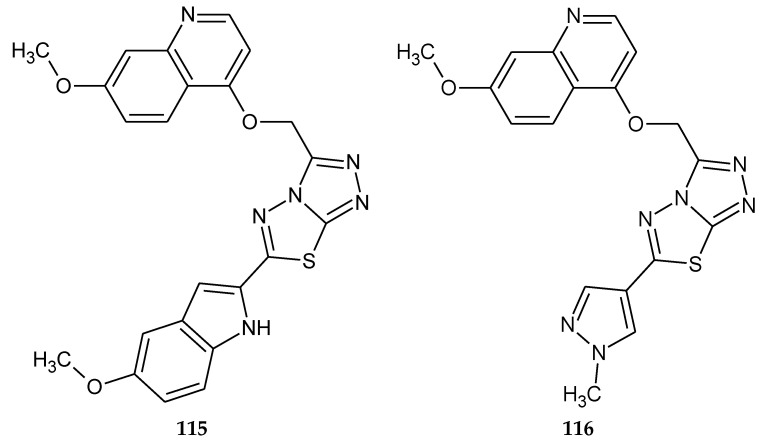
The structures of 6-(5-methoxy-1*H*-indol-2-yl)-3-(((7-methoxyquinolin-4-yl)oxy)methyl)-[1,2,4]triazolo[3,4-b][1,3,4]thiadiazole (**115**) and 3-(((7-methoxyquinolin-4-yl)oxy)methyl)-6-(1-methyl-1H-pyrazol-4-yl)-[1,2,4]triazolo[3,4-b][1,3,4]thiadiazole (**116**).

In 2018, Zhang and co-workers described anticancer activity a series of [1,2,4]triazolo[3,4-b][1,3,4]thiadiazole derivatives. For anticancer investigations authors used SNU-5 (human gastric cancer), MKN45 (human gastric cancer) and EBC-1 (human lung cancer) cell lines. Compound **115** (Figure 63) exhibited high antiproliferative activities against EBC-1 cancer cell line with IC_50_ value 85.6 nM. The most active compound **116** (Figure 63) has the IC_50_ value of 88 nM relative to the human gastric cancer cell line and it is selective towards c-Met kinase and exhibits over a 2500-fold selective inhibition of 16 tyrosine kinases used in presented investigations [70].

**Figure 64:**
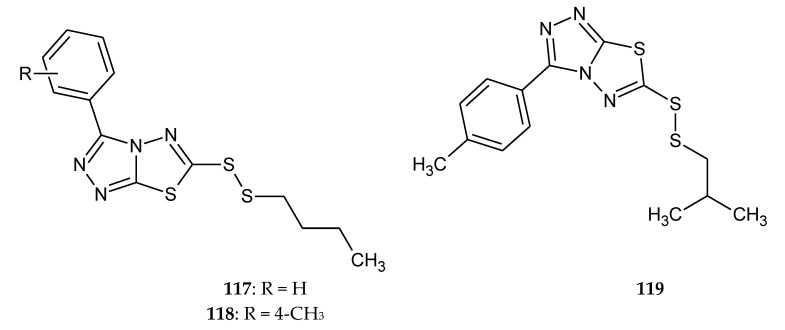
The structure of the most active compounds 3-phenyl-6-(*n*-butyldisulfanyl)-1,2,4-triazole-[3,4-b]-1,3,4-thiadiazole (**117**), 3-(4-methylphenyl)-6-(*n*-butyldisulfanyl)-1,2,4-triazole-[3,4-b]-1,3,4-thiadiazole (**118**), 3-(4-methylphenyl)-6-(isobutyldisulfanyl)-1,2,4-triazole-[3,4-b]-1,3,4-thiadiazole (**119**).

Liu et al. (2019) presented synthesis of twenty three 3,6-disubstituted 1,2,4-triazolo[3,4-b]-1,3,4-thiadiazole derivatives and their antiproliferative activities in vitro against human hepatocarcinoma (SMMC-7721), HeLa, human lung carcinoma (A549), and mouse fibroblasts (L929) cell lines by the CCK-8 assay. The bioassay results demonstrated that all tested compounds exhibited antiproliferation with different degrees, and some compounds **117**–**119** (Figure 64) showed better effects than reference drug 5-Fluorouracil [71].

### 3.2. Imidazo-Thiadiazole Hydrids

**Figure 65:**
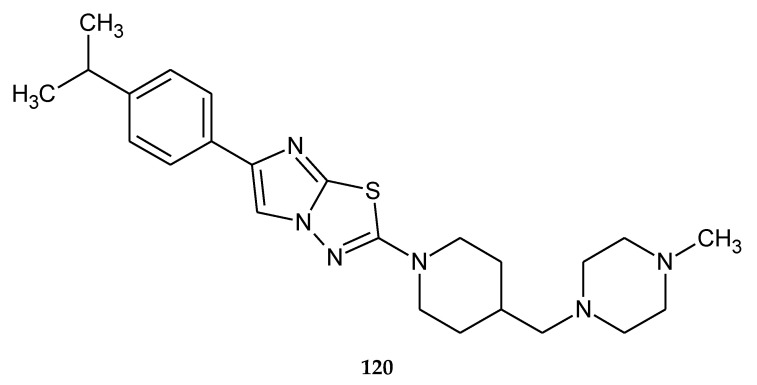
The structure of 2-(4-isopropylphenyl)-6-{[4-(4-methylpiperazin)methyl]piperidin} imidazo[2,1-b][1,3,4]thiadiazol (**120**).

In 2010, Nir and co-workers patented synthesis and anticancer evaluation of a new compounds prepared by condensation of 1-(*tert*-butoxycarbonyl)piperidine-4-carboxylic acid with 1-methylpiperazine. The most active compound **120** (Figure 65) has shown growth-inhibiting activities with EC_50_ value of 3–4 μM for tumor HCT116 cell line (human colon cancer) [72].

**Figure 66:**
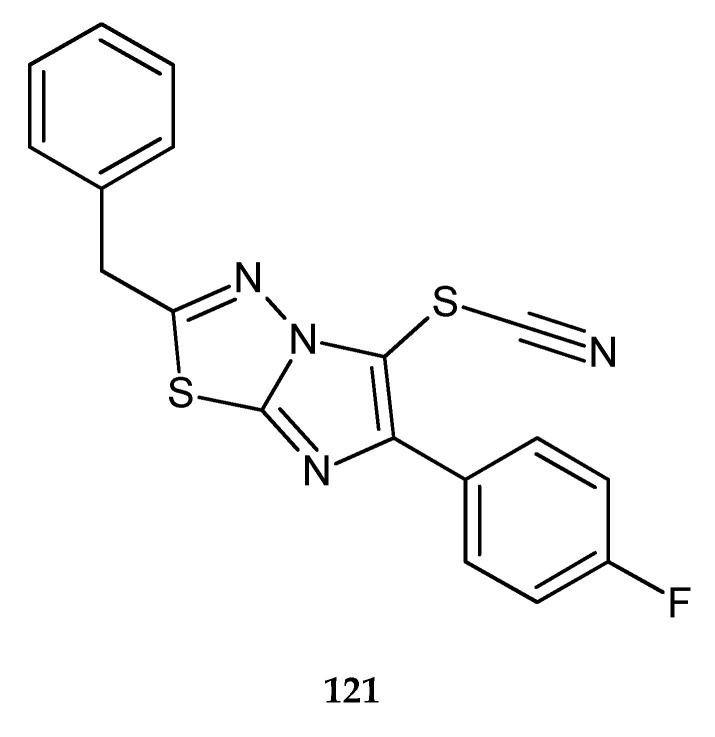
The structure of 2-benzyl-6-(4-fluorophenyl)imidazo[2,1-b][1,3,4]thiadiazol-5-yl thiocyanate (**121**).

A series of new imidazo[2,1-b]thiadiazoles were synthesized by Karki et al. (2011). The compounds demonstrated inhibition of the growth leukemia cell lines. Compound **121** has shown the best anticancer activity (Figure 66) with the IC_50_ value of 8 μM. FACS (fluorescence-activated cell sorting) analysis, in conjunction with mitochondrial membrane potential and DNA fragmentation studies, has demonstrated that this compound induced apoptosis without cell cycle arrest [73].

**Figure 67:**
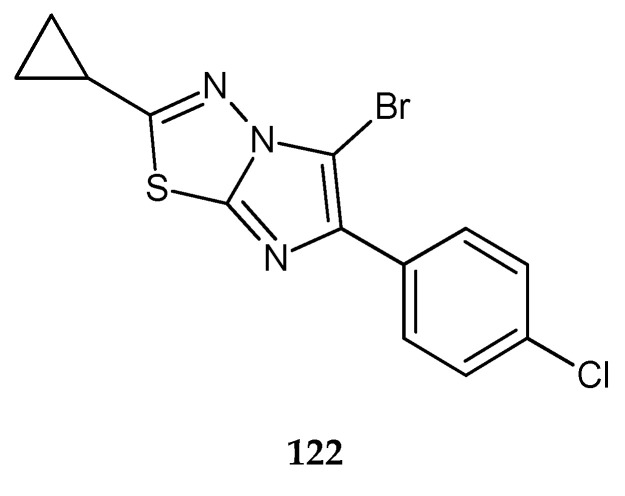
The structure of 5-bromo-6-(4-chlorophenyl)-2-cyclopropylimidazo[2,1-b][1,3,4]thiadiazole (**122**).

In 2011, Song and co-workers reported synthesis and anticancer evaluation of a series of compounds with 1,3,4-thiadiazole heterocyclic rings. Seven compounds have shown good anticancer activity at a single high dose (10^-5^ M) in full NCI 60 cell panel studies. 5-Bromo-6-(4-chlorophenyl)-2-cyclopropylimidazo[2,1-b][1,3,4]thiadiazole (**122**) (Figure 67) indicated the best growth-inhibiting activities against leukemic cancer cell line [74].

**Figure 68:**
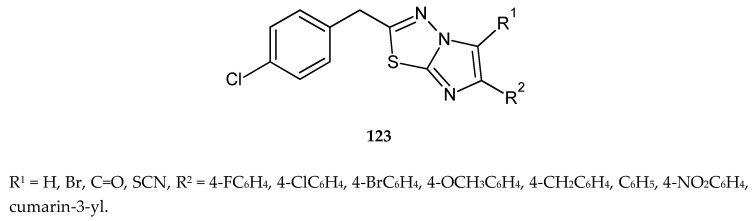
The structures of 2-(4-chlorobenzyl)-6-arylimidazo[2,1-b][1,3,4]thiadiazoles (**123**).

In 2014, Kumar and co-workers described synthesis and anticancer activity in a series of 2-(4-chlorobenzyl)-6-arylimidazo[2,1-b][1,3,4]thiadiazoles (**123**) (Figure 68). The compounds were evaluated for their anticancer activity against human and murine cancer cell lines. 2-(4-Chlorobenzyl)-6-(2-oxo-2*H*-chromen-3-yl)imidazo[2,1-b][1,3,4]thiadiazole-5-carboaldehyde and 2-(4-chlorobenzyl)-6-(2-oxo-2H-chromen-3-yl)imidazo[2,1-b][1,3,4]thiadiazol-5-yl were most potent against all cell lines [75].

**Figure 69:**
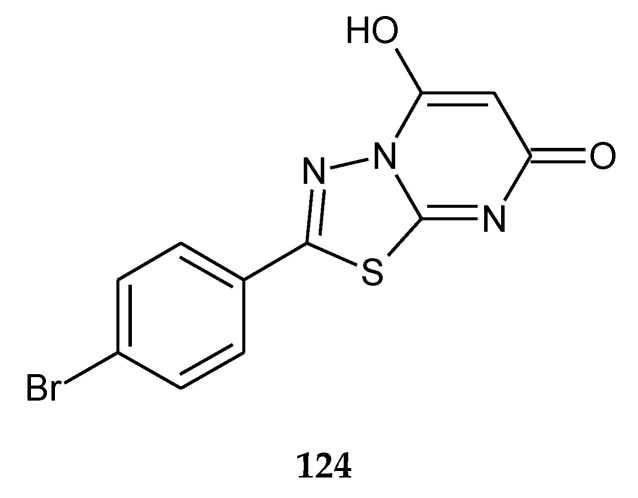
The structure of 2-(4-bromophenyl)-5-hydroxy-7*H*-1,3,4-thiadiazolo[3,2-a]pyrimidin-7-one (**124**).

**Figure 70:**
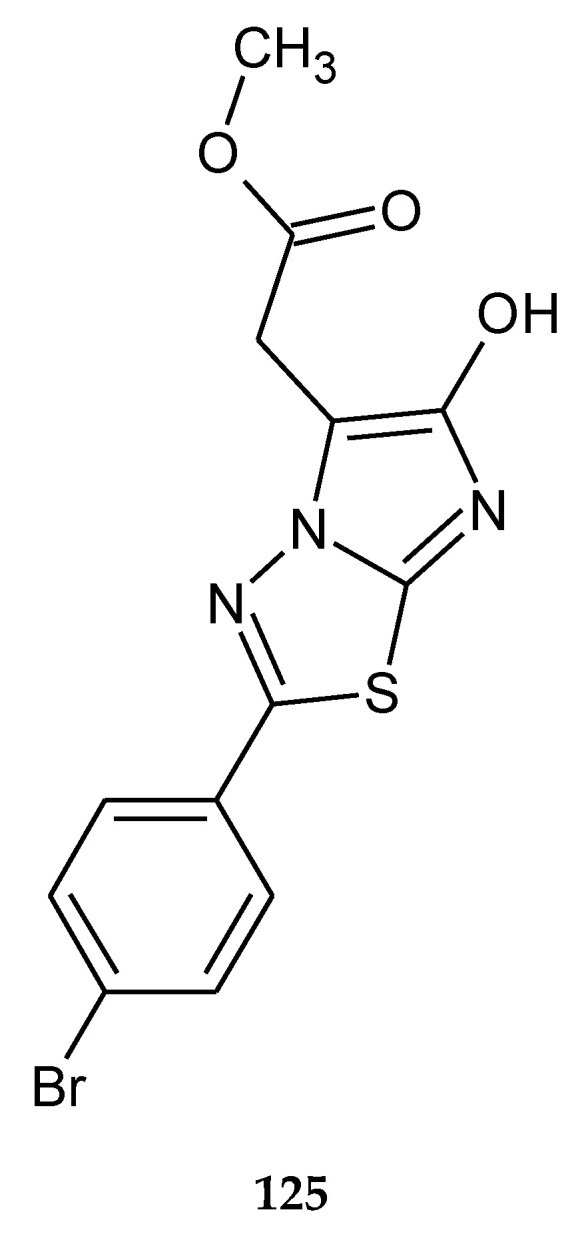
The structure of methyl 2-[2-(4-bromophenyl)-6-hydroxyimidazo[2,1-b]-1,3,4-thiadiazol-5-yl]acetate (**125**).

In 2014, the novel 1,3,4-thiadiazole analogues with expected anticancer activity were reported by Abdel Rahman et al. The compounds were evaluated for their anticancer activity against A549 (human lung carcinoma) cell line using sulforhodamine B assay. The majority of the compounds have shown good cytotoxicity but lower than the reference drug doxorubicin. The best IC_50_ value was 2.58 μM for compound **124** (Figure 69), but one hybrid imidazole–thiadiazole showed good activity with IC_50_ value 4.74 μM (**102**) (Figure 70). The authors performed a docking study in order to find a molecular target for new compounds. All compounds shared some binding interactions with fibroblast stromelysin-1 similar to those of the native ligand inhibitor (PUN-142372). This fact suggests that investigated compounds might possibly act as fibroblast stromelysin-1 inhibitors [76].

**Figure 71:**
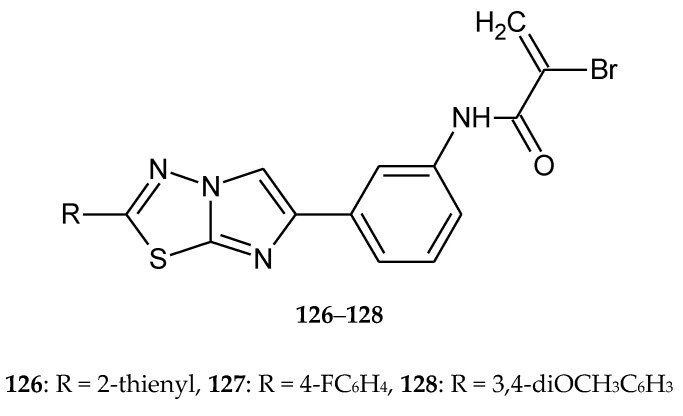
The structures of 2-bromo-*N*-{3-[2-(2-thienyl)imidazo[2,1-b][1,3,4]thiadiazol-6-yl]phenyl}acrylamide (**126**), 2-bromo-*N*-{3-[2-(4-fluorophenyl)imidazo[2,1-b][1,3,4]thiadiazol-6-yl]phenyl}acrylamide (**127**), 2-bromo-*N*-{3-[2-(3,4-dimethoxyphenyl)imidazo[2,1-b][1,3,4]thiadiazol-6-yl]phenyl}acrylamide (**128**).

A series of heterobivalent hybrids with a 1,3,4-thiadiazole ring were obtained by Romagnoli and co-workers in 2015. The compounds were evaluated for their anticancer activity against L1210 (murine leukemia), FM3A (murine mammary carcinoma), human T-lymphoblastoid (CEM) and human cervix carcinoma (HeLa) cell lines. Most of the tested compounds have shown good antiproliferative activity, with IC_50_ values: 0.17–0.67, 0.25–0.87, 0.042–0.61 μM, respectively (**126**–**128**) (Figure 71). The authors observed that selected compounds induced apoptosis, which was connected with the release of cytochrome c and cleavage of multiple caspases [77].

**Figure 72:**
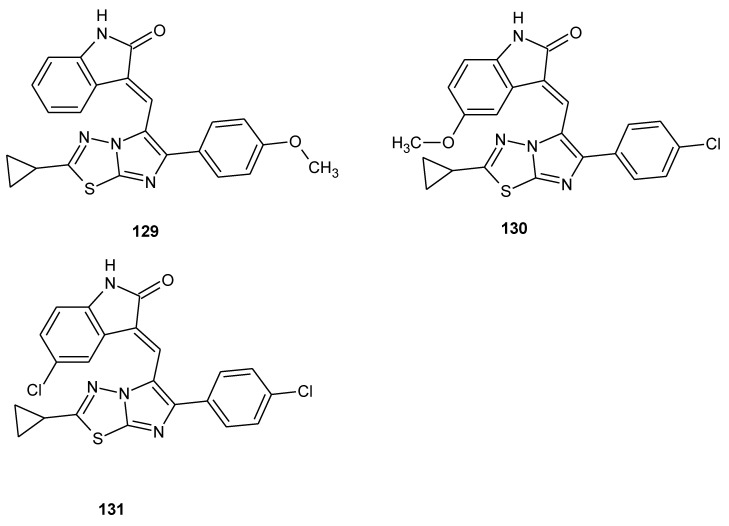
The structures of (*E*)-3-((6-(4-chlorophenyl)-2-cyclopropylimidazo[2,1-b][1,3,4]thiadiazol-5-yl)methylene)-5-methoxyindolin-2-one (**129**), (*E*)-3-((2-cyclopropyl-6-(4-methoxyphenyl)imidazo[2,1-b][1,3,4]thiadiazol-5-yl)methylene)indolin-2-one (**130**) and (*E*)-5-chloro-3-((6-(4-chlorophenyl)-2-cyclopropylimidazo[2,1-b][1,3,4]thiadiazol-5-yl)methylene)indolin-2-one (**131**).

The design, synthesis and biological evaluation of imidazo[2,1-b][1,3,4]thiadiazole-indolin-2-one conjugates were reported by Narashika et al. in 2018. All new compounds were investigated for antiproliferative activity in different human cancer cell lines. The compounds have shown good anticancer activity during in vitro tests, with GI_50_ values from 0.13 to 3.8 μΜ. Next, the conjugates were evaluated for cell cycle analysis, tubulin polymerization assay and apoptosis. The three most active conjugates (**129**–**131**) (Figure 72) were induced in accumulation of cells in G2/M phase, disruption of microtubule network and inhibition of tubulin assembly. The same compounds showed apoptosis in HeLa cell line. Docking studies showed that presented compounds bind with αAsn101, αThr179, αSer178, βCys241, βLys254 and βLys352 in the colchicine-binding site of the tubulin [78].

## 4. Conclusions

In conclusion, this paper gives an overview of cytotoxic properties of various 1,3,4-thiadiazole derivatives. There are definitely more reports on simple 2,5-disubstituted derivatives of 1,3,4-thiadiazole than condensed systems of thiadiazole with other rings. It seems to be a very promising group of chemical compounds. Some of them have promising anticancer activity, higher than reference drugs (Tables S1 and S2 Supplementary Materials). Both cytotoxic and cytostatic properties have been confirmed on various cell lines. Analyzing the results, it can be concluded that more active compounds are among the simple derivatives. Some of them contain an important pharmacophore group in their structure —the trifluoromethyl substituent.

## Supplementary Materials

The following are available online. Table S1: Cytotoxic activity of the most active 2,5-disubstituted-1,3,4-thiadiazole. Table S2: Cytotoxic activity of the most active 1,3,4-thiadiazole condensed with other heterocyclic rings. Table S3: The Table of abbreviations.
