# Supplementary material for: Cytotoxic Properties of 1,3,4-Thiadiazole Derivatives—A Review
Source: Molecules. 2020 Sep 20;25(18):4309. doi: 10.3390/molecules25184309 (PMC7570754; doi:10.3390/molecules25184309)
Supplement: Supplementary file 1 [file molecules-25-04309-s001.pdf]

Supplementary Materials

Table S1 Cytotoxic activity of the most active 2,5-disubstituted-1,3,4-thiadiazole.

Table S2 Cytotoxic activity of the most active 1,3,4-thiadiazole condensed with other heterocyclic rings.

Table S3 The Table of abbreviations.

Table S1 Cytotoxic activity of the most active 2,5-disubstituted-1,3,4-thiadiazole.

Compounds<br><br>Molecular formula	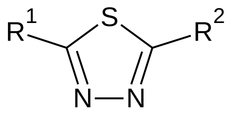		IC_{50} value for tested compounds [µM]<br>IC_{50} value for reference drugs [µM]							
	R^1	R^2	HL-60	HepG2	MCF-7	A549	PC3	MDA	HCT-116	HT-29
**16**<br><br>$C_{14}H_{18}F_3N_7S$	CF_3	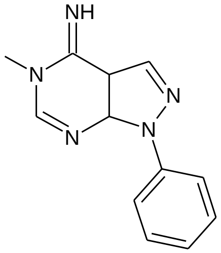	0.08<br>0.55^b	nt	nt	nt	nt	nt	nt	nt

**17**<br>$C_{20}H_{12}F_3N_7S$	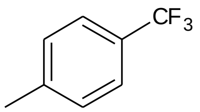	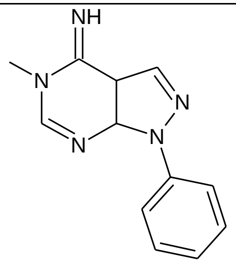	0.21<br>0.55[b]	nt	nt	nt	nt	nt	nt	nt
**31**<br>$C_{19}H_{16}F_3N_3O_2S_2$	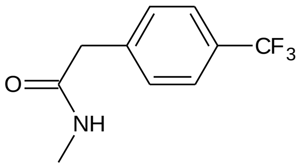	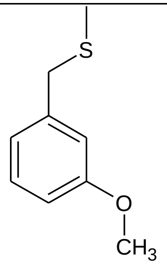	nt	nt	nt	nt	130	9<br>20[c]	nt	nt
**43**<br>$C_9H_8ClN_3S_3$	NH_2	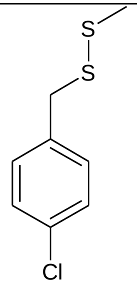	nt	nt	1.78	4.04<br>19.90[d]	nt	nt	nt	nt
**58**<br>$C_{29}H_{33}FN_40_9S_3$	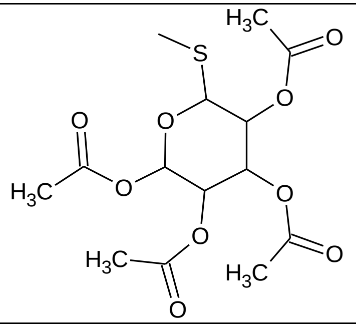	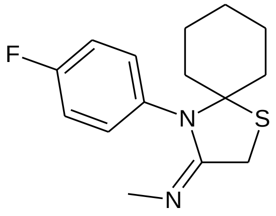	nt	0.18<br>0.1171[b]	nt	nt	0.121<br>0.219[b]	nt	0.092<br>0.126<br>b	nt

**62**<br>$C_{20}H_{15}N_5O_5S_2$	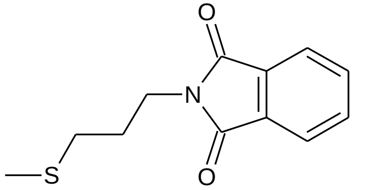	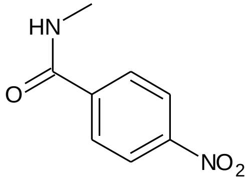	nt	nt	23.83	nt	nt	nt	nt	27.21
**63**<br>$C_{10}H_8FN_30S_2$	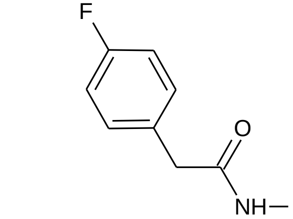	SH	nt	nt	nt	nt	64.46	nt	nt	33.67
**84**<br>$C_{21}H_{20}F_3N_3OS_4$	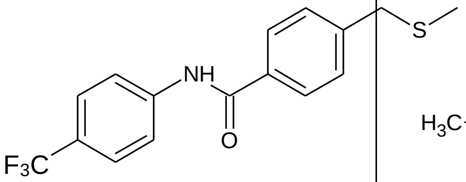	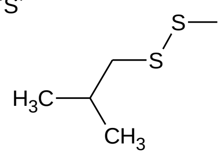	nt	nt	nt	3.81<br>8.13[d]	nt	nt	nt	nt
**85**<br>$C_{20}H_{21}N_3OS_4$	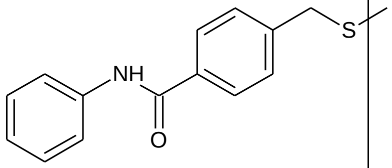	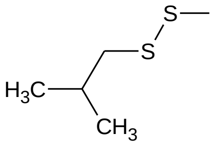				2.12<br>8.13[d]				
**86**<br>$C_{20}H_{20}BrN_3OS_4$	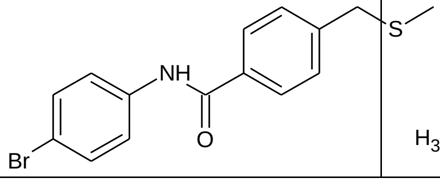	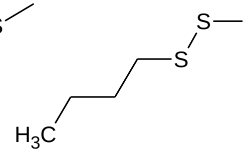				3.43<br>8.13[d]				

HL-60 - human leukemia; HepG2 - liver cancer; MCF-7 - breast cancer; A549 - human lung carcinoma, PC3 - prostate cancer; MDA - breast cancer; HCT-116 - human colorectal carcinoma; HT-29 – colon cancer
[a]bleomycyna, [b]doxorubicyna, [c]imatynib, [d]5-fluorouracil, [e]cisplatin; nt – not tested

Table S2 Cytotoxic activity of the most active 1,3,4-thiadiazole condensed with other heterocyclic rings.

Compounds<br><br>Molecular formula	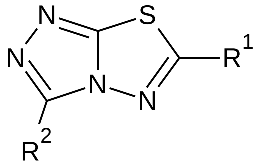		IC$_{50}$ value for tested compounds [µM]<br><br>IC$_{50}$ value for reference drugs [µM]			
	R^1	R^2	HepG2	A549	SMMC-7721	MCF
**106**<br><br>$C_{23}H_{15}FN_6OS$	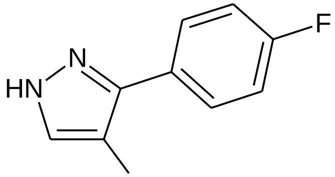	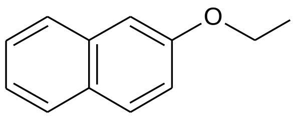	1.134<br><br>34.96[b]	nt	nt	nt
**113-1**<br><br>$C_{14}H_{16}N_4S_3$	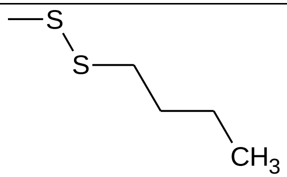	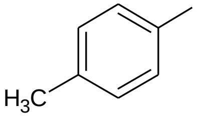	nt	6.35<br><br>8.13[d]	3.89<br><br>5.62[d]	4.49<br><br>14.26 [d]
**113-2**<br><br>$C_{13}H_{14}N_4S_3$	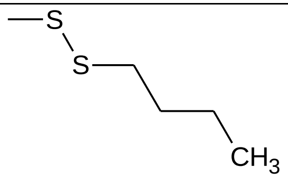	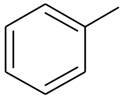	nt	18.73<br><br>8.13[d]	1.93<br><br>5.62[d]	8.20<br><br>14.26[d]

**113-3**<br><br>$C_{14}H_{16}N_4S_3$	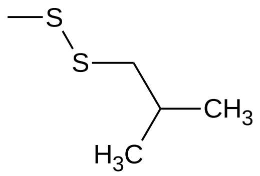	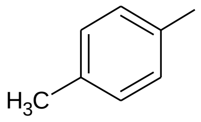	nt	9.85<br>8.13[d]	4.39<br>5.62[d]	9.66<br>14.26[d]

HL-60 - human leukemia; HepG2 - liver cancer; MCF-7 - breast cancer; A549 - human lung carcinoma, PC3 - prostate cancer; MDA - breast cancer; HCT-116 - human colorectal carcinoma; HT-29 – colon cancer

[a]bleomycyna, [b]doxorubicyna, [c]imatynib, [d]5-fluorouracil, [e]cisplatin; nt - not tested

Table S3 Table of abbreviations

abbreviation	expansion
A549	lung cancer cell line
A875	human melanoma cell line
ADM	adriamycin
B16-F10	melanoma cell lines
Beas-2B	human bronchial epithelial cell lines
BGC823	gastric cancer cell line
BT474	breast cancer cell line
BxPC-3	human pancreatic cancer cell line
C6	Glioma cell line
CAI	Carbonic anhydrase I
CAII	Carbonic anhydrase II
CAIX	Carbonic anhydrase IX
Caski	human cervical carcinoma cell lines
CAXII	Carbonic anhydrase XII
CCK-8 assay	cell counting Kit-8 (CCK-8) allows convenient assays
CCRF-CEM	leukemia human cancer cell lines
CEM	human T-lymphoblastoid cell lines
CHO	Chinese hamster ovary cell line
CHO-K1	normal Chinese hamster ovary cancer cell line
DBA/2	mouse lymphoma cell lines
DHFR	Dihydrofolate reductase

DU145	human prostate cancer cell line
EAC	Ehrlichs Ascites Carcinoma cells
EBC-1	human lung cancer cell lines
EC_{50}	half maximal effective concentration
ELISA assays	enzyme linked immunosorbent assays
ERK1/2	extracellular signal-regulated kinases
FACS analysis	fluorescence-activated cell sorting
FAK	Focal Adhesion Kinase assay
FM3A	murine mammary carcinoma cell lines
FTC- 238	thyroid carcinoma cell line
G1	Gap 1 phase
GI_{50}	the concentration causing 50% cell growth inhibition
H1975	human lung adenocarcinoma cell line
H460	human lung cancer cell line
HCT116	human colon cancer cell line
HCT116	human colorectal adenocarcinoma cell line
HCT15	colon cancer cell line
HCT15	colon adenocarcinoma cell lines
HEK 293	epidermal kidney cell line
HELA	cervical cancer cell line
HEPG2	human hepatocellular carcinoma cell lines
HGC27	gastric cancer cell lines
HL60	human leukemia cell line

HT-29	colon carcinom cell line
HT-29	using human colon cancer cells
Huh-7	human hepatocellular carcinoma cell lines
IC_{50}	half maximal inhibitory concentration
ISL	indisulam
K562	Bcr-Abl positive cell line
K562	myelogenous leukemia cell lines
L1210	mouse leukemia cell line
L1210	lymphocytic leukemia cancer cell lines
L1210	murine leukemia cancer cell lines
L929	normal cell line
L929	mouse fibroblasts
MCF-7	human breast cancer cell line
MDA	Breast cancer cell lines
MDA-MB-23	breast cancer cell lines
MKN45	human gastric cancer cell lines
MOGGCCM	brain astrocytoma cell line
MOLT-4	human acute T lymphoblastic leukemia cell lines
MRC-5	normal fibroblasts cell line
MTT	colorimetric assay for assessing cell metabolic activity. MTT- tetrazolium dye.
NCI 60	cancer cell line panel used by the National Cancer Institute (NCI)
NCI-H226	lung cancer cell line
P19	Teratoma cell line

PANC1	human pancreatic cancer cell lines
PC3	tumor human prostate cell line
PUN-142372	fibroblast stromelysin-1 similar to those of the native ligand inhibitor
S	synthesis Phase
SaOS-2	human osteosarcoma cell lines
SAR analysis	structure–activity relationship
SiHa	human cervical cancer cell lines
SK-MEL-2	skin cancer cell line
SKNMC	neuroblastoma cell line
SKOV-3	human ovarian carcinoma cell line
SK-OV-3	ovarian cancer cell line
SMMC-7721	hepatocarcinoma cell lines
Snu5	gastric carcinoma cells lines
SRB assay	sulforhodamine B (SRB) Assay
SW1116	human colorectal carcinoma cell lines
T47D	breast carcinoma cell line
TE671	rhabdomyosarcoma/medulloblastoma cell line
U87	Glioblastoma cell lines
UACC-257	melanoma human cancer cell lines
